# The Individual and Combined Effects of Prenatal Micronutrient Supplementations on Neurobehavioral Developmental Disorders in Preschool Children

**DOI:** 10.3390/children12050602

**Published:** 2025-05-05

**Authors:** Liwen Ding, Esben Strodl, Maolin Zhang, Weiqing Chen

**Affiliations:** 1 Department of Epidemiology and Health Statistic, School of Public Health, Sun Yat-sen University, Guangzhou 510080, China; 2 School of Psychology and Counselling, Queensland University of Technology, Brisbane, QLD 4059, Australia; 3 School of Health Management, Xinhua College, Sun Yat-sen University, Guangzhou 510080, China

**Keywords:** neurobehavioral development, prenatal micronutrient, multivitamin supplementation, interaction, preschool children, crossover analysis

## Abstract

**Background:** Neurobehavioral developmental disorders significantly affect children’s future well-being and contribute to the global disease burden. While prenatal micronutrient supplementation is crucial for fetal neural development, their individual and combined effects on subsequent neurobehavioral outcomes in childhood remain poorly understood. This study aimed to examine the individual and combined effects of prenatal micronutrient supplementation on neurobehavioral developmental disorders in preschool children, and to explore their effects across specific developmental domains. **Methods:** 15,636 mother-child dyads were recruited from the 2022 children’s survey in Shenzhen, China. Mothers provided information on prenatal supplementation of calcium, folic acid, iron, and multivitamins. Five domains of children’s neurobehavioral functioning were assessed using the Ages and Stages Questionnaire-Third Edition (communication, gross motor, fine motor, problem-solving, and personal-social status). Logistic regression models were used to estimate the effect of micronutrient supplementations on NDDs across crude, adjusted, and full-inclusion models. Combined effects were assessed by multiplicative and additive interactions calculated from crossover analysis. **Results:** 11.7% of preschool children were identified as at risk for neurobehavioral developmental disorders, with the highest prevalence in the gross motor domain. Prenatal multivitamin supplementation showed a protective effect against neurobehavioral developmental disorders (OR = 0.73, 95% CI = 0.66–0.81). Interaction analysis revealed that the combination of iron and multivitamins further enhanced this protection, with both multiplicative (IOR = 1.26, 95% CI = 1.02–1.57) and additive interactions (RERI = 0.18, 95% CI = 0.02–0.35). The problem-solving domain consistently showed the greatest benefit from the supplementation of these micronutrients individually and in combination. **Conclusions:** Prenatal multivitamin supplementation reduces the risk of neurobehavioral developmental disorders, especially when combined with iron supplementation. These findings highlight the potential benefits of prenatal co-supplementation strategies to improve neurobehavioral outcomes in offspring. Further studies are recommended to confirm these findings and explore underlying mechanisms.

## 1. Introduction

Neurobehavioral development is the process by which the brain and nervous system develop, influencing behavior, cognition, and emotional regulation [1]. In children, this development is crucial for future educational attainment, mental health, career success, and overall quality of life [2]. However, neurobehavioral development disorders remain prevalent worldwide, affecting core functions such as perception, motor skills, and language [3]. According to a World Health Organization (WHO) report, up to 58 million children (7%) worldwide experienced developmental disorders in 2019, with a neurobehavioral disorder being the most common, accounting for 58.6% of children with a developmental disorder [4]. In China, the prevalence of these disorders among children ranges from 3.2% to 13.9% [5,6,7,8]. In severe cases, such disorders may manifest as overt conditions like autism spectrum disorder (ASD) and attention deficit hyperactivity disorder (ADHD), both of which are increasingly prevalent worldwide [9,10,11]. These conditions impose significant burdens on individuals, families, and society. For individuals, ASD accounts for over 691.5 disability-adjusted life years (DALYs) per 100,000 population globally, ranking among the top 10 neurological conditions [12]. For families, it adds $3020 in annual medical costs and leads to a significant productivity loss for parents [13]. Societal lifetime costs can reach up to $3.2 million per individual, including healthcare, education, and productivity losses [14]. Therefore, early intervention is crucial to reduce the risk of more severe neurodevelopmental conditions and to alleviate these long-term burdens.

The prenatal period is a critical period for neurobehavioral development when interventions are most effective in reducing the risk and severity of neurodevelopmental disorders [15,16]. Prenatal nutrition, especially micronutrients, plays a vital role in fetal neural development with long-term health implications [17]. However, deficiencies in key micronutrients are prevalent among pregnant women worldwide, including in China, with increasing trends [18,19,20]. Evidence suggests that iron intake may improve neurobehavioral outcomes in children [21,22], although this finding has not been consistent [23]. Similarly, while a randomized controlled trial (RCT) indicated that vitamin D benefits motor development [24], other research found no such effect [25,26]. Likewise, some studies suggest that iodine supplementation supports cognitive development in children [27,28], yet a systematic review of RCTs reported little to no impact on neurobehavioral development [29]. These inconsistencies may arise from heterogeneity in study design, including confounding biases and variations in neurobehavioral assessment methods [25,26]. Thus, there is a need for large sample studies with well-controlled confounders and sensitive, reliable neurobehavioral assessment tools to address these disparities. The Ages & Stages Questionnaire Third Edition (ASQ-3) is a well-validated and standardized tool that comprehensively assesses children’s language, motor, cognitive, and social development. Compared to other neurodevelopmental assessments, its low cost, time efficiency, and reliance on parent report make it highly suitable for large-scale assessment [30]. Therefore, ASQ-3 provides a practical and reliable tool for neurobehavioral assessment.

During pregnancy, micronutrient requirements increase, and most expectant mothers supplement with multiple micronutrients concurrently [31]. These micronutrients may interact in complex ways. For instance, folic acid and vitamin B12 are involved in homocysteine metabolism and DNA methylation, with synergistic effects on neural tube development [32,33]. In contrast, certain metal ions, such as copper and zinc, exhibit antagonistic effects due to competition for shared transport proteins (e.g., metallothioneins), potentially increasing the risk of neurological disorders [34,35]. Therefore, the combined effects of micronutrients on the neurobehavioral development of offspring may change by such interactions. However, most existing research has focused on the effects of individual micronutrients, rather than on their combined effects. A population study found an interaction between folic acid and vitamin B12 related to cognitive performance; however, the study focused on adults over 60 years old [36]. Given the unique plasticity and vulnerability of the developing brain during the prenatal period, such findings have limited reference for early neurodevelopment [15,16]. In pregnant women, studies on micronutrient interactions have primarily focused on short-term maternal or fetal outcomes, such as gestational diabetes, preeclampsia, and preterm birth [37,38,39,40]. Nevertheless, they do not capture the long-term neurodevelopmental consequences in offspring, which has a more direct impact on children’s future health [2]. Our previous research showed that combining folic acid with multivitamins or iron reduced the risk of childhood obesity compared to individual supplementation [41]. However, research on the effects of micronutrient interactions on neurobehavioral development in children remains limited.

Therefore, this study aimed to examine both the individual and combined effects of micronutrient supplementation during pregnancy on neurobehavioral development in preschool children, and to further explore their specific effects across the five developmental domains using the ASQ-3.

## 2. Materials and Methods

### 2.1. Participants

**Figure 1:**
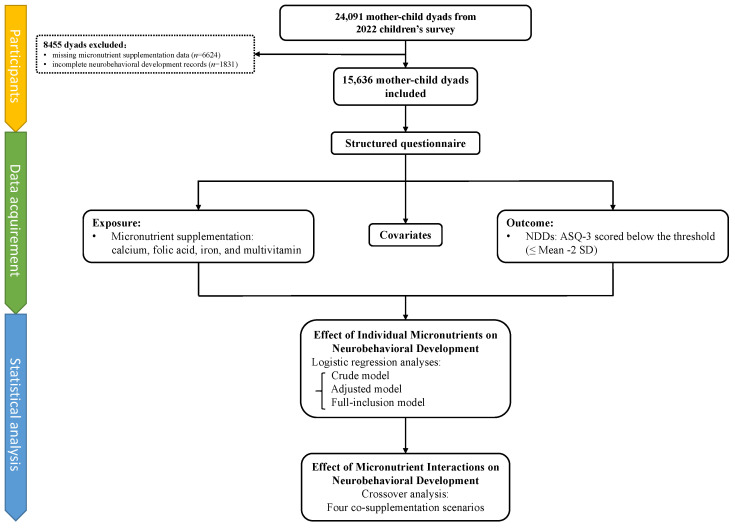
Study profile. ASQ-3, Age and Developmental Progress Questionnaire; NDDs, neurobehavioral developmental disorders.

Participants were recruited from the 2022 children’s survey on neurobehavioral development conducted in 235 kindergartens in Longhua District, Shenzhen, China, which enrolled 24,091 mother–child dyads. After exclusion of cases with missing micronutrient supplementation data (*n* = 6624) and incomplete neurobehavioral development records (*n* = 1831), a total of 15,636 participants were included, see Figure 1. As detailed in Supplementary Text S1, this sample size meets the minimum requirement for this study [42]. This study was approved by the Ethics Committee of the School of Public Health, Sun Yat-sen University. Informed consent was obtained from all children’s primary guardians.

### 2.2. Data Acquisition

Data were collected between March 2022 and April 2023 through a self-administered online structured questionnaire, completed by children’s mothers under the supervision of childcare practitioners and kindergarten teachers. The questionnaire has been used in annual children’s surveys since 2014, with clarity and readability validated, and has been widely used in numerous studies [41,43,44,45,46]. It contained demographic characteristics, maternal condition during pregnancy (e.g., micronutrient supplementation, pregnancy complications, health behaviors), current parental lifestyle and health condition (e.g., smoking, drinking, diseases), neonatal birth characteristics (e.g., birth weight, preterm birth, delivery mode), and current children health condition (e.g., neurobehavioral development, nutritional condition). Detailed information and coding are provided in Supplementary Table S1.

### 2.3. Prenatal Micronutrient Supplementation

Micronutrient supplementation during pregnancy (calcium, folic acid, iron, and multivitamin) was assessed through maternal self-reported responses to four separate questions: “Did you take calcium/folic acid/iron/multivitamins during your pregnancy?” Responses were recorded as ‘NO’ or ‘YES’, with “NO” serving as the reference.

### 2.4. Outcome

The neurobehavioral development in preschool children was assessed by the Ages and Stages Questionnaire-Third Edition (ASQ-3). The ASQ-3 is a sensitive and reliable tool that has been validated across various countries, including China, and is suitable for children aged 1–66 months [47,48,49]. It assesses five domains of neurobehavioral function: communication, gross motor, fine motor, problem-solving, and personal social status. The score for each domain was classified into three levels: (1) Above the threshold (>mean − 1 standard deviation [SD]), indicating age-appropriate development; (2) Close to the threshold (mean − 2 SD to mean − 1 SD), requiring further monitoring; and (3) Below the threshold (≤mean − 2 SD). In our study, the presence of neurobehavioral developmental disorders (NDDs) was identified when at least one domain scored below the threshold, while neurobehavioral developmental normality (NDN) was defined by all domains being above the threshold or close to it [6,50].

### 2.5. Covariates

Based upon previous research [21,27,51,52,53] and the univariate and multivariate analyses conducted on our dataset (see Supplementary Table S2), the covariates included in our analyses were the child’s basic characteristics (age, sex, birth season, residence type), maternal demographic characteristics (education, household income, age of conception, pre-pregnancy BMI), pregnancy and perinatal characteristics (intrauterine growth restriction [IUGR], parity, preterm birth [PTB], and birth weight [BW]), and childhood family environment (parental depression, family functioning, feeding pattern). These covariates had an average missing data rate of 3.9% (range: 0–12.9%), with missing data imputed using multiple imputations through Predictive Mean Matching (PMM) [54].

### 2.6. Statistical Analysis

In the descriptive analysis, all variables were compared between NDDs and NDN groups using one-way ANOVA and *t*-test for continuous variables and chi-square test for categorical variables. The main inferential statistical analyses were conducted in two parts, as outlined below.

#### 2.6.1. Individual Effects of Micronutrients on Neurobehavioral Development

We conducted univariate and multivariate logistic regression analyses under three different models to improve the reliability and robustness of the results [55,56,57]: the crude model, without adjustment for confounders; the adjusted model, adjusted for selected confounders; and the full-inclusion model, including all micronutrients and adjusting for all confounders to address the confounding effects of co-supplementation.

#### 2.6.2. Combined Effects of Micronutrients on Neurobehavioral Development

To examine the combined effects of micronutrient supplementation, a crossover analysis was performed under the three models [58,59]. This method is widely utilized in studies exploring interaction effects in health outcomes [41,60,61]. Participants were categorized into four exposure groups based on co-supplementation scenarios, with no supplementation of either micronutrient as the reference group. Multiplicative and additive interactions were estimated through regression models that included these micronutrients and their interaction terms, with results presented as Interaction Odds Ratio (IOR) for multiplicative interactions, Relative Excess Risk due to Interaction (RERI), and Attributable Proportion due to Interaction (AP) for additive interactions, as well as Odds Ratios (ORs) for each exposure group. To further identify the domains of micronutrient effects, we also analyzed these interactions across the five neurobehavioral domains.

All statistical analyses above were performed via R version 4.2.3, with two-sided *p*-values < 0.05 considered statistically significant.

## 3. Results

### 3.1. Participants’ Characteristics

Table 1 presents the characteristics and micronutrient supplementation status of the study participants, comparing those with NDDs and NDN. The mean age was 4.6 ± 0.6 years for the children, and 34.0 ± 5.5 years for their mothers. The average birth weight of children was 3.1 ± 0.6 kg. Among the 15,636 mother–child dyads analyzed, child’s sex and birth season were evenly distributed across groups. More than half of the children were Shenzhen residents, 0.89% had experienced IUGR, 7.2% were born preterm, and approximately half were breastfeeding. Most mothers had at least a high school education, over half had a household income exceeding RMB 20,000, and nearly 70% had a normal pre-pregnancy BMI. There were 12.7% of parents reporting depression and nearly 40% of families classified as dysfunctional. For micronutrient supplementation, calcium (75.8%) and folic acid (88.2%) were prevalent among the participants, whereas iron (46.0%) and multivitamins (44.4%) were less common.

The total mean score of ASQ-3 was 277.5 ± 26.4, with the domain means ranging from 51.7 ± 10.2 in the fine motor domain to 57.6 ± 5.4 in the communication domain. Most of the children scored within the normal group in each domain. The highest prevalence of NDDs was observed in the gross motor domain (8.88%), while the problem-solving domain had the lowest prevalence (0.70%). Overall, 11.7% of children were identified as at risk for NDDs (see Table 2).

### 3.2. Individual Effects of Micronutrients on Neurobehavioral Development

**Figure 2:**
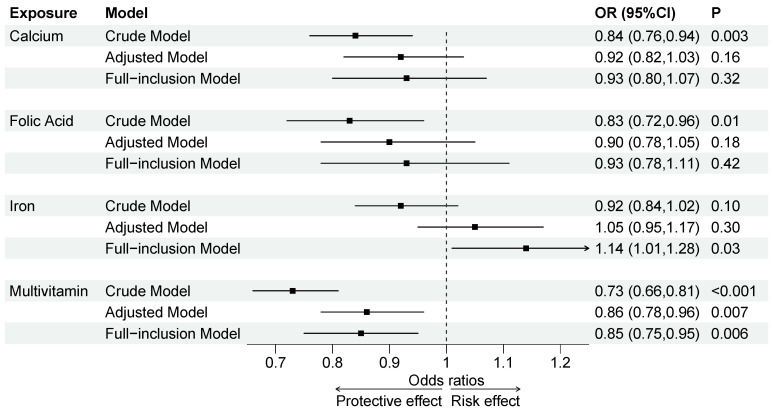
Individual effects of micronutrients on NDDs in crude, adjusted and full-inclusion models. Adjusted Model: Adjusted for child’s basic characteristics, maternal demographic characteristics, pregnancy and perinatal characteristics and childhood family environment. Full-inclusion Model: Included all micronutrients in the model and adjusted for child’s basic characteristics, maternal demographic characteristics, pregnancy and perinatal characteristics and childhood family environment. OR, odds ratio; CI, confidence interval; P, *p*-value.

In the crude model, compared to the reference group, supplementation with calcium (OR = 0.84, 95% CI = 0.76–0.94), folic acid (OR = 0.83, 95% CI = 0.72–0.96), or multivitamins (OR = 0.73, 95% CI = 0.66–0.81) was negatively associated with NDDs, whereas no significant associations were found for iron. In the adjusted and full-inclusion models, only multivitamin supplementation remained significantly associated with a decreased risk of NDDs (Adjusted Model: OR = 0.86, 95% CI = 0.78–0.96; Full-inclusion Model: OR = 0.85, 95% CI = 0.75–0.95) (see Figure 2 and Supplementary Table S3).

In the communication domain, calcium (OR = 0.64, 95% CI = 0.47–0.88) and multivitamins (OR = 0.61, 95% CI = 0.45–0.84) exhibited the strongest protective effects. In the problem-solving domain, folic acid (OR = 0.53, 95% CI = 0.33–0.85) and iron (OR = 0.59, 95% CI = 0.40–0.88) showed the highest protective effects. Notably, in the fine motor domain, only multivitamins (OR = 0.81, 95% CI = 0.67–0.99) were negatively associated with NDDs in the crude model (see Supplementary Table S4).

### 3.3. Combined Effects of Micronutrients on Neurobehavioral Development

In the crude model, the combination of calcium and folic acid supplementation during pregnancy (OR = 0.81, 95% CI = 0.69–0.95), as well as the combination of calcium and iron supplementation (OR = 0.83, 95% CI = 0.74–0.94), showed an increased protective effect on NDDs compared to individual calcium or iron supplementation. Additionally, the combination of folic acid and iron supplementation (OR = 0.80, 95% CI = 0.68–0.93) showed a stronger protective effect than individual folic acid supplementation. Similarly, the combination of folic acid and multivitamin supplementation (OR = 0.68, 95% CI = 0.58–0.80) showed a stronger effect than individual multivitamin supplementation. Notably, the combination of iron and multivitamin supplementation (OR = 0.75, 95% CI = 0.67–0.85) was associated with increased protective effect on NDDs compared to individual multivitamin supplementation, with significant multiplicative (IOR = 1.26, 95% CI = 1.02–1.57) as well as additive interactions (RERI = 0.18, 95% CI = 0.02–0.35) observed (see Table 3). Compared to individual calcium or multivitamin supplementation, the combination of calcium and multivitamin supplementation showed an increased protective effect across all models (Crude model: OR = 0.71, 95% CI = 0.62–0.80; Adjusted model: OR = 0.85, 95% CI = 0.74–0.98; Full-inclusion model: OR = 0.82, 95% CI = 0.69–0.97), although no significant additive or multiplicative interactions were observed (see Supplementary Table S5).

**Figure 3:**
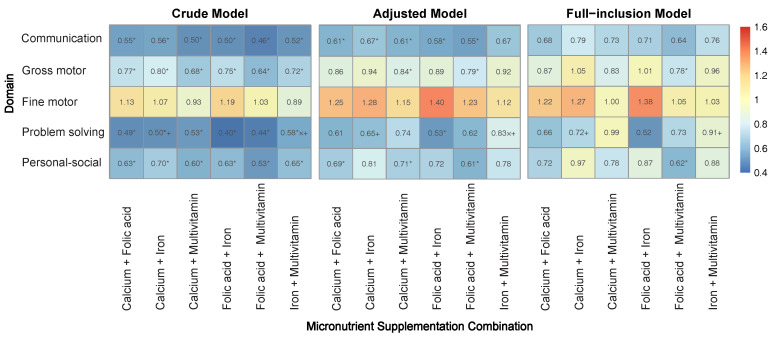
Heatmap of combined effects of micronutrients on NDDs across five domains in crude, adjusted and full-inclusion models. Adjusted Model: Adjusted for child’s basic characteristics, maternal demographic characteristics, pregnancy and perinatal characteristics and childhood family environment. Full-inclusion Model: Included all micronutrients in the model and adjusted for child’s basic characteristics, maternal demographic characteristics, pregnancy and perinatal characteristics and childhood family environment. * *p* < 0.05. × significant multiplicative interactions. + significant additive interactions.

The combined effects of micronutrients on neurobehavioral development across five domains of neurobehavioral function are presented in Figure 3. Protective effects were observed for the problem-solving, communication, personal-social, and gross motor domains, while the fine motor domain showed no significant effect. The combinations of calcium with folic acid, calcium with iron, and folic acid with iron were most protective in the problem-solving domain, whereas the combinations of calcium with multivitamins, folic acid with multivitamins, and iron with multivitamins were most protective in the communication domain. Specifically, the combination of folic acid and iron demonstrated the strongest protective effect in the problem-solving domain across all models (Crude Model: OR = 0.40, 95% CI = 0.24–0.68; Adjusted Model: OR = 0.53, 95% CI = 0.31–0.92; Full-inclusion Model: OR = 0.52, 95% CI = 0.25–1.07).

Significant multiplicative or additive interactions were observed only in the problem-solving domain. In the crude model, there were both multiplicative and additive interactions for iron and multivitamins (IOR = 2.90, 95% CI = 1.18–7.63; RERI = 0.68, 95% CI = 0.24–1.11), and additive interactions for calcium and iron (RERI = 0.68, 95% CI = 0.17–1.18). In the adjusted model, the combination of iron and multivitamins maintained both multiplicative (IOR = 2.6, 95% CI = 1.06–6.88) and additive interactions (RERI = 0.68, 95% CI = 0.11–1.25), whereas calcium and iron had only additive interactions (RERI = 0.75, 95% CI = 0.20–1.31). In the full-inclusion model, significant additive interactions were observed for both the combination of iron and multivitamins (RERI = 0.67, 95% CI = 0.05–1.28) and calcium and iron (RERI = 0.72, 95% CI = 0.09–1.34) (see Supplementary Table S5).

## 4. Discussion

In our study, we found that 11.7% of preschool children were identified as at risk for NDDs, with the highest prevalence of neurodevelopmental disorders being in the gross motor domain. Prenatal multivitamin supplementation was identified as a protective factor against NDDs across the crude, adjusted and full-inclusion models. When examining the combined effects of micronutrients on neurobehavioral development, our results indicated the combination of iron and multivitamins significantly increased the protective effect of multivitamins alone on NDDs, with significant multiplicative and additive interactions. Further analysis by neurobehavioral domain showed that micronutrient co-supplementation influenced problem-solving, communication, personal-social, and gross motor functions. In the problem-solving domain, the combination of iron and multivitamins showed both multiplicative and additive interactions, and calcium and iron exhibited an additive interaction.

Our findings align with previous research showing that multivitamin supplementation is associated with a reduced risk of NDDs [62,63]. Other studies have also suggest that individual supplementation, such as vitamins D and B12, is associated with a lower risk of NDDs [25,64,65,66]. Vitamins play a crucial role in fetal brain development, acting as cofactors in neurotransmitter synthesis and enzymatic metabolism processes [67]. For example, vitamin B12 is essential in fatty acid metabolism required for myelin sheath production, while vitamin B6 serves as a coenzyme in the production of various amino acid neurotransmitters, both of which may affect neurobehavioral development [68,69]. Similarly, vitamin A derivatives, such as retinoids, regulate neuronal differentiation and have been implicated in functions like memory and sleep [70]. Notably, research suggested that multivitamins may have broader effects on neurobehavioral development, as they can act on multiple biological pathways [69]. However, some studies have discovered that multivitamins do not always have a greater effect on cognitive function than single vitamins [71]. Thus, the role of multivitamins versus single-vitamin supplementation in neurobehavioral development warrants further investigation. Despite the importance of vitamins in neurobehavioral development, our study found that less than half of pregnant women took multivitamins. Numerous surveys have also revealed insufficient vitamin supplementation during pregnancy to meet recommended levels [66], emphasizing the need to prioritize vitamin supplementation during pregnancy to reduce the risk of NDDs in their offspring.

Folic acid is crucial for neural tube development, and its supplementation was more prevalent than the supplementation of other micronutrients in our study, likely due to its recommendation in the WHO’s essential drug list for pregnant women [72,73,74]. The research linking folic acid supplementation on neurobehavioural developmental is complex. Some studies have found a positive correlation between maternal folic acid status and children’s neurobehavioral development [75,76]. In contrast, others have reported a u-shaped curve, with low and high doses of folic acid supplementation increasing the risk of ASD and food allergies [77,78]. Given this risk, it is important to precisely define appropriate levels of folic acid supplementation, as folic acid has been officially recommended for pregnant mothers [66].

Iron and calcium, because of their roles in neurotransmitter, energy metabolism and myelin formation, may also affect neurobehavioral development [79,80]. Although no significant associations were observed between calcium or iron supplementation and the risk of NDDs in our study. Similar non-significant findings have been reported in other studies, potentially due to variations in study populations, limited statistical power, measurement errors, or other confounding factors [81,82].

When analyzing the individual effects of micronutrients on NDDs, the study found that calcium and folic acid had a protective effect on NDDs in the crude model. However, this effect disappeared after adjusting for other micronutrients, whereas the protective effect of multivitamins remained significant both before and after adjustment. These results suggest that the effect of calcium or folic acid on NDDs may develop from their interaction with multivitamins. This hypothesis was further supported by our subsequent analysis, which showed that co-supplementation with calcium and multivitamins was associated with a lower risk of NDDs compared to supplementation with either calcium or multivitamins alone. One explanation for this co-supplementation benefit is that the vitamin D in multivitamins promotes calcium absorption and metabolism, supporting calcium’s role in nerve conduction and neurotransmitter release [83,84]. Similar benefits have been observed in thyroid hormone production and musculoskeletal development [85,86]. However, no significant multiplicative or additive interactions between calcium and multivitamins were found in our study, probably because of limitations in the modeling and testing methods. Future studies using advanced techniques, such as Bayesian modeling, could provide more precise estimates of these interactions’ effects on neurobehavioral development [87,88].

Although our study did not demonstrate a greater protective effect of co-supplementation with iron and vitamins compared to iron alone, consistent with findings from a previous RCT [89], it did show an enhanced protective effect compared to multivitamin supplementation alone, with significant multiplicative and additive interactions. This enhanced effect is likely attributable to the fact that vitamins C, A, and B enhance iron absorption, storage, and transport, and contribute to neurotransmitter synthesis, thereby promoting neurobehavioral development [90,91,92]. Given its potential benefits, co-supplementation of iron and vitamins has attracted increasing attention globally. Indeed, it is now incorporated into nutrient supplementation programs in several countries [93,94].

Among the five neurobehavioral domains, most of the significant micronutrient interactions were observed in the problem-solving and communication domains, especially with the combination of iron and multivitamins. These domains may be more dependent on micronutrient-related neurotransmitters, while the gross and fine motor domains have relatively stable neurotransmitter requirements, and the personal-social domain is influenced more by hormonal and environmental factors [95,96].

There are potential limitations of this study that need to be considered when interpreting its results. First, the generalizability of the results may be limited by the sample only from Shenzhen, China, as dietary habits may vary in other regions. Second, the use of a structured questionnaire for data collection may have introduced recall bias. The ASQ-3 was used to assess children’s neurobehavioral development, and it may be subject to reporting bias by the mothers compared to standard clinical diagnoses. Additionally, maternal micronutrient intake was assessed via binary responses rather than specific dosages. Finally, despite controlling for confounders, this study is based on observational data, so the findings reflect correlations rather than causality. Therefore, further research with diverse populations and more detailed micronutrient data is needed to provide more comprehensive evidence and explore the biological mechanisms underlying these interactions.

## 5. Conclusions

In summary, our study found that prenatal multivitamins supplementation is significantly associated with a reduced risk of NDDs in preschool children, with the strongest protective effect observed in the problem-solving domain. Additionally, the combination of iron and multivitamins further enhanced this protective effect, with both significant multiplicative and additive interactions. These findings underscore the importance of adequate micronutrient supplementation during pregnancy in their offspring, especially the potential benefits of co-supplementation. With a large-scale sample and a standardized measurement tool, this research provides evidence for dietary interventions during the critical prenatal period for the early prevention of specific domain-related NDDs, thereby promoting normal neurobehavioral development in children. Despite the promising results, future trials with detailed clinical data are necessary to confirm the combined effects of prenatal micronutrient supplementation and its underlying mechanisms.

## Figures and Tables

**Table 1:** Basic characteristics of study participants in the 2022 children’s survey.

Characteristic	Overall (*n* = 15,636) ^1^	NDDs (*n* = 13,804) ^1^	NDN (*n* = 1832) ^1^	*p*-Value ^2^
Child’s age	4.6 ± 0.6	4.6 ± 0.6	4.6 ± 0.5	<0.001
Child’s sex				<0.001
Male	8346 (53.4%)	7219 (52.3%)	1127 (61.5%)	
Female	7290 (46.6%)	6585 (47.7%)	705 (38.5%)	
Birth season				<0.001
Spring	4225 (27.0%)	3746 (27.1%)	479 (26.1%)	
Summer	4482 (28.7%)	3845 (27.9%)	637 (34.8%)	
Autumn	2945 (18.8%)	2618 (19.0%)	327 (17.8%)	
Winter	3984 (25.5%)	3595 (26.0%)	389 (21.2%)	
Residence type				<0.001
Shenzhen residents	9415 (60.2%)	8507 (61.6%)	908 (49.6%)	
Non-Shenzhen residents	6221 (39.8%)	5297 (38.4%)	924 (50.4%)	
Maternal education				<0.001
Less than high school	1614 (10.3%)	1262 (9.14%)	352 (19.2%)	
High school and higher	14,022 (89.7%)	12,542 (90.9%)	1480 (80.8%)	
Household income				<0.001
<RMB 20,000	7314 (46.8%)	6262 (45.4%)	1052 (57.4%)	
≥RMB 20,000	8322 (53.2%)	7542 (54.6%)	780 (42.6%)	
Maternal conception age	34.0 ± 5.5	34.0 ± 5.5	33.7 ± 5.7	0.028
Pre-pregnancy BMI				<0.001
BMI < 18.5	2890 (18.5%)	2550 (18.5%)	340 (18.6%)	
18.5 ≤ BMI < 24	10,571 (67.6%)	9392 (68.0%)	1179 (64.4%)	
BMI ≥ 24	2175 (13.9%)	1862 (13.5%)	313 (17.1%)	
Intrauterine growth retardation				<0.001
No	15,497 (99.1%)	13,697 (99.2%)	1800 (98.3%)	
Yes	139 (0.89%)	107 (0.78%)	32 (1.75%)	
Parity				0.21
No	8810 (56.3%)	7752 (56.2%)	1058 (57.8%)	
Yes	6826 (43.7%)	6052 (43.8%)	774 (42.2%)	
Preterm birth				<0.001
No	14,517 (92.8%)	12,851 (93.1%)	1666 (90.9%)	
Yes	1119 (7.2%)	953 (7.0%)	166 (9.1%)	
Child’s birth weight	3.1 ± 0.6	3.1 ± 0.6	3.0 ± 0.7	<0.001
Parental depression				<0.001
No	13,652 (87.3%)	12,140 (87.9%)	1512 (82.5%)	
Yes	1984 (12.7%)	1664 (12.1%)	320 (17.5%)	
Family function				<0.001
Normal	9697 (62.0%)	8772 (63.5%)	925 (50.5%)	
Dysfunction	5939 (38.0%)	5032 (36.5%)	907 (49.5%)	
Feeding pattern				<0.001
Breastfeeding	8803 (56.3%)	7815 (56.6%)	988 (53.9%)	
Formula feeding	1665 (10.6%)	1415 (10.3%)	250 (13.6%)	
Mixed feeding	5168 (33.1%)	4574 (33.1%)	594 (32.4%)	
Calcium supplementation				0.003
No	3781 (24.2%)	3286 (23.8%)	495 (27.0%)	
Yes	11,855 (75.8%)	10,518 (76.2%)	1337 (73.0%)	
Folic acid supplementation				0.013
No	1846 (11.8%)	1597 (11.6%)	249 (13.6%)	
Yes	13,790 (88.2%)	12,207 (88.4%)	1583 (86.4%)	
Iron supplementation				0.11
No	8451 (54.0%)	7428 (53.8%)	1023 (55.8%)	
Yes	7185 (46.0%)	6376 (46.2%)	809 (44.2%)	
Multivitamin supplementation				<0.001
No	8690 (55.6%)	7549 (54.7%)	1141 (62.3%)	
Yes	6946 (44.4%)	6255 (45.3%)	691 (37.7%)	
Probiotic intake				0.010
No	3098 (19.8%)	2777 (20.1%)	321 (17.5%)	
Yes	12,538 (80.2%)	11,027 (79.9%)	1511 (82.5%)	

^1^ Data are presented as Mean ± SD or N(%). ^2^ *p*-value was based on one-way analysis of means and Pearson’s Chi-squared test where appropriate.

**Table 2:** Neurobehavioral development across 5 domains in the 2022 children’s survey.

Domains	Score, Mean ± SD	Prevalence, N (%)
Communication	58 ± 5.4	
Normal		15,457 (98.9%)
Delay		179 (1.14%)
Gross motor	54 ± 8.6	
Normal		14,248 (91.1%)
Delay		1388 (8.88%)
Fine motor	52 ± 10.2	
Normal		15,214 (97.3%)
Delay		422 (2.70%)
Problem-solving	57 ± 5.7	
Normal		15,526 (99.3%)
Delay		110 (0.70%)
Personal-social	57 ± 5.6	
Normal		15,281 (97.7%)
Delay		355 (2.27%)
Total	277 ± 26.4	
Normal		13,804 (88.3%)
Delay		1832 (11.7%)

**Table 3:** Combined effect of micronutrients on NDDs in the crude model.

Micronutrients		N (%)	OR	IOR	RERI	AP
Calcium	Folic acid					
No	No	210 (13.5%)	1.00 (ref)	—	—	—
No	Yes	39 (13.3%)	0.98 (0.67, 1.40)	—	—	—
Yes	No	285 (12.8%)	0.94 (0.77, 1.14)	—	—	—
Yes	Yes	1298 (11.2%)	**0.81 (0.69, 0.95)**	0.88 (0.60, 1.32)	−0.11 (−0.49, 0.28)	−0.13 (−0.60, 0.34)
Calcium	Iron					
No	No	457 (13.2%)	1.00 (ref)	—	—	—
No	Yes	38 (12.3%)	0.93 (0.64, 1.30)	—	—	—
Yes	No	566 (11.4%)	**0.85 (0.74, 0.97)**	—	—	—
Yes	Yes	771 (11.2%)	**0.83 (0.74, 0.94)**	1.06 (0.74, 1.56)	0.06 (−0.28, 0.40)	0.07 (−0.34, 0.49)
Calcium	Multivitamin					
No	No	423 (13.3%)	1.00 (ref)	—	—	—
No	Yes	718 (13.0%)	0.98 (0.86, 1.12)	—	—	—
Yes	No	72 (12.1%)	0.90 (0.68, 1.17)	—	—	—
Yes	Yes	619 (9.7%)	**0.71 (0.62, 0.80)**	0.80 (0.60, 1.08)	−0.17 (−0.44, 0.10)	−0.25 (−0.62, 0.13)
Folic acid	Iron					
No	No	233 (13.8%)	1.00 (ref)	—	—	—
No	Yes	16 (10.5%)	0.74 (0.42, 1.22)	—	—	—
Yes	No	790 (11.7%)	**0.83 (0.71, 0.97)**	—	—	—
Yes	Yes	793 (11.3%)	**0.80 (0.68, 0.93)**	1.30 (0.78, 2.33)	0.23 (−0.18, 0.63)	0.29 (−0.23, 0.81)
Folic acid	Multivitamin					
No	No	235 (14.1%)	1.00 (ref)	—	—	—
No	Yes	906 (12.9%)	0.91 (0.78, 1.06)	—	—	—
Yes	No	14 (8.0%)	**0.53 (0.29, 0.90)**	—	—	—
Yes	Yes	677 (10.0%)	**0.68 (0.58, 0.80)**	1.41 (0.82, 2.61)	0.24 (−0.08, 0.56)	0.36 (−0.13, 0.84)
Iron	Multivitamin					
No	No	795 (13.4%)	1.00 (ref)	—	—	—
No	Yes	346 (12.6%)	0.93 (0.81, 1.06)	—	—	—
Yes	No	228 (9.1%)	**0.64 (0.55, 0.75)**	—	—	—
Yes	Yes	463 (10.5%)	**0.75 (0.67, 0.85)**	**1.26 (1.02, 1.57)**	**0.18 (0.02, 0.35)**	**0.24 (0.02, 0.46)**

## Data Availability

The data supporting the findings of this study are available on request from the corresponding author. The data are not publicly available due to privacy or ethical restrictions.

## Supplementary Materials

The following supporting information can be downloaded at: https://www.mdpi.com/article/10.3390/children12050602/s1, Supplementary Table S1: Detailed information and coding of data collected in the 2022 children’s survey. Supplementary Table S2: Univariate and multivariate analysis of covariates. Supplementary Table S3: Individual effects of micronutrients on NDDs in the crude, adjusted and full-inclusion model. Supplementary Table S4: Individual effects of micronutrients on NDDs across five domains in the crude, adjusted and full-inclusion model. Supplementary Table S5: Combined effects of micronutrients on NDDs across five domains in the crude, adjusted and full-inclusion model. Supplementary Text S1: Sample Size Calculation.

## Abbreviations

The following abbreviations are used in this manuscript:

ADHD	Attention Deficit Hyperactivity Disorder
AP	Attributable Proportion due to Interaction
ASQ-3	Age and Developmental Progress Questionnaire
ASD	Autism Spectrum Disorder
BMI	Body Mass Index
BW	Birth Weight
CDE	Controlled Direct Effect
DALYs	Disability-Adjusted Life Years
IOR	Interaction Odds Ratio
IUGR	Intrauterine Growth Restriction
NDDs	Neurobehavioral Developmental Disorders
NDN	Neurobehavioral Developmental Normality
OR	Odds Ratio
PMM	Predictive Mean Matching
PTB	Preterm Birth
RCT	Randomized Controlled Trial
RERI	Relative Excess Risk due to Interaction
SD	Standard Deviation
WHO	World Health Organization

## References

[1] Shah P.J., Boilson M., Rutherford M., Prior S., Johnston L., Maciver D., Forsyth K. (2022). Neurodevelopmental disorders and neurodiversity: Definition of terms from Scotland’s National Autism Implementation Team. Br. J. Psychiatry.

[2] Grantham-McGregor S., Cheung Y.B., Cueto S., Glewwe P., Richter L., Strupp B. (2007). Developmental potential in the first 5 years for children in developing countries. Lancet.

[3] Thapar A., Cooper M., Rutter M. (2017). Neurodevelopmental disorders. Lancet. Psychiatry.

[4] WHO Global Report on Children with Developmental Disabilities: From the Margins to the Mainstream. https://www.unicef.org/documents/global-report-children-developmental-disabilities.

[5] Zhang J., Lu H., Sheng Q., Zang E., Zhang Y., Yuan H., Chen B., Tang W. (2024). The Influence of Perinatal Psychological Changes on Infant Neurodevelopment in Shanghai, China: A Longitudinal Group-based Trajectory Analysis. J. Affect. Disord..

[6] Ma R., Wang P., Yang Q., Zhu Y., Zhang L., Wang Y., Sun L., Li W., Ge J., Zhu P. (2024). Interpregnancy interval and early infant neurodevelopment: The role of maternal-fetal glucose metabolism. BMC Med..

[7] Li Y., Chen X., Shang X., He H. (2022). Developmental Screening and Analysis of Influencing Factors in 2,980 Infants Under 3 Months in Beijing. Beijing Med..

[8] Chen C., Lin Y., Yan W., Zhang Y. (2022). Ages and stages questionnaire in screening developmental levels of infants from 6 to 12 months and risk factors analysis. J. Bio-Educ..

[9] Maenner M.J., Warren Z., Williams A.R., Amoakohene E., Bakian A.V., Bilder D.A., Durkin M.S., Fitzgerald R.T., Furnier S.M., Hughes M.M. (2023). Prevalence and Characteristics of Autism Spectrum Disorder Among Children Aged 8 Years - Autism and Developmental Disabilities Monitoring Network, 11 Sites, United States, 2020. Morb. Mortal. Wkly. Report. Surveill. Summ..

[10] Bachmann C.J., Scholle O., Bliddal M., dosReis S., Odsbu I., Skurtveit S., Wesselhoeft R., Vivirito A., Zhang C., Scott S. (2024). Recognition and management of children and adolescents with conduct disorder: A real-world data study from four western countries. Child Adolesc. Psychiatry Ment. Health.

[11] Jensen de López K.M., Thirup Møller H. (2024). Prevalence of Autism in Scandinavian Countries (Denmark, Norway, Sweden), and Nordic Countries (Finland, Iceland, the Faroe Islands, and Greenland). Neuropsychiatr. Dis. Treat..

[12] Collaborators G.N.S.D. (2024). Global, regional, and national burden of disorders affecting the nervous system, 1990-2021: A systematic analysis for the Global Burden of Disease Study 2021. Lancet. Neurol..

[13] Lavelle T.A., Weinstein M.C., Newhouse J.P., Munir K., Kuhlthau K.A., Prosser L.A. (2014). Economic burden of childhood autism spectrum disorders. Pediatrics.

[14] Rogge N., Janssen J. (2019). The Economic Costs of Autism Spectrum Disorder: A Literature Review. J. Autism Dev. Disord..

[15] Lewis A.J., Galbally M., Gannon T., Symeonides C. (2014). Early life programming as a target for prevention of child and adolescent mental disorders. BMC Med..

[16] McGowan E.C., Hofheimer J.A., O’Shea T.M., Kilbride H., Carter B.S., Check J., Helderman J., Neal C.R., Pastyrnak S., Smith L.M. (2022). Analysis of Neonatal Neurobehavior and Developmental Outcomes Among Preterm Infants. JAMA Netw. Open.

[17] Cusick S.E., Barks A., Georgieff M.K., Andersen S.L. (2022). Nutrition and Brain Development. Sensitive Periods of Brain Development and Preventive Interventions.

[18] Hu Y., Wang R., Mao D., Chen J., Li M., Li W., Yang Y., Zhao L., Zhang J., Piao J. (2021). Vitamin D Nutritional Status of Chinese Pregnant Women, Comparing the Chinese National Nutrition Surveillance (CNHS) 2015-2017 with CNHS 2010-2012. Nutrients.

[19] Black R.E., Victora C.G., Walker S.P., Bhutta Z.A., Christian P., de Onis M., Ezzati M., Grantham-McGregor S., Katz J., Martorell R. (2013). Maternal and child undernutrition and overweight in low-income and middle-income countries. Lancet.

[20] Han T., Dong J., Zhang J., Zhang C., Wang Y., Zhang Z., Xiang M. (2022). Nutrient supplementation among pregnant women in China: An observational study. Public Health Nutr..

[21] Arija V., Hernández-Martínez C., Tous M., Canals J., Guxens M., Fernández-Barrés S., Ibarluzea J., Babarro I., Soler-Blasco R., Llop S. (2019). Association of Iron Status and Intake During Pregnancy with Neuropsychological Outcomes in Children Aged 7 Years: The Prospective Birth Cohort Infancia y Medio Ambiente (INMA) Study. Nutrients.

[22] Janbek J., Sarki M., Specht I.O., Heitmann B.L. (2019). A systematic literature review of the relation between iron status/anemia in pregnancy and offspring neurodevelopment. Eur. J. Clin. Nutr..

[23] Moumin N.A., Shepherd E., Liu K., Makrides M., Gould J.F., Green T.J., Grzeskowiak L.E. (2024). The Effects of Prenatal Iron Supplementation on Offspring Neurodevelopment in Upper Middle- or High-Income Countries: A Systematic Review. Nutrients.

[24] Wicklow B., Gallo S., Majnemer A., Vanstone C., Comeau K., Jones G., L’Abbe M., Khamessan A., Sharma A., Weiler H. (2016). Impact of Vitamin D Supplementation on Gross Motor Development of Healthy Term Infants: A Randomized Dose-Response Trial. Phys. Occup. Ther. Pediatr..

[25] Mutua A.M., Mogire R.M., Elliott A.M., Williams T.N., Webb E.L., Abubakar A., Atkinson S.H. (2020). Effects of vitamin D deficiency on neurobehavioural outcomes in children: A systematic review. Wellcome Open Res..

[26] McCarthy E.K., Murray D.M., Malvisi L., Kenny L.C., J O.B.H., Irvine A.D., Kiely M.E. (2018). Antenatal Vitamin D Status Is Not Associated with Standard Neurodevelopmental Assessments at Age 5 Years in a Well-Characterized Prospective Maternal-Infant Cohort. J. Nutr..

[27] Markhus M.W., Dahl L., Moe V., Abel M.H., Brantsæter A.L., Øyen J., Meltzer H.M., Stormark K.M., Graff I.E., Smith L. (2018). Maternal Iodine Status is Associated with Offspring Language Skills in Infancy and Toddlerhood. Nutrients.

[28] Murcia M., Espada M., Julvez J., Llop S., Lopez-Espinosa M.J., Vioque J., Basterrechea M., Riaño I., González L., Alvarez-Pedrerol M. (2018). Iodine intake from supplements and diet during pregnancy and child cognitive and motor development: The INMA Mother and Child Cohort Study. J. Epidemiol. Community Health.

[29] Jalali Chimeh F., Aghaie E., Ghavi S., Fatahnia R. (2024). Investigation of the Effects of Maternal Nutrition during Pregnancy on Cognitive Functions of Toddlers: A Systematic Review. Int. J. Prev. Med..

[30] Squires J., Bricker D.D., Twombly E. (2009). Ages & Stages Questionnaires, Third Edition (ASQ-3): A Parent-Completed Child Monitoring System.

[31] Devakumar D., Fall C.H., Sachdev H.S., Margetts B.M., Osmond C., Wells J.C., Costello A., Osrin D. (2016). Maternal antenatal multiple micronutrient supplementation for long-term health benefits in children: A systematic review and meta-analysis. BMC Med..

[32] Bergen N.E., Schalekamp-Timmermans S., Jaddoe V.W., Hofman A., Lindemans J., Russcher H., Tiemeier H., Steegers-Theunissen R.P., Steegers E.A. (2016). Maternal and Neonatal Markers of the Homocysteine Pathway and Fetal Growth: The Generation R Study. Paediatr. Perinat. Epidemiol..

[33] Kok D.E., Dhonukshe-Rutten R.A., Lute C., Heil S.G., Uitterlinden A.G., van der Velde N., van Meurs J.B., van Schoor N.M., Hooiveld G.J., de Groot L.C. (2015). The effects of long-term daily folic acid and vitamin B12 supplementation on genome-wide DNA methylation in elderly subjects. Clin. Epigenetics.

[34] Krężel A., Maret W. (2017). The Functions of Metamorphic Metallothioneins in Zinc and Copper Metabolism. Int. J. Mol. Sci..

[35] Oestreicher P., Cousins R.J. (1985). Copper and zinc absorption in the rat: Mechanism of mutual antagonism. J. Nutr..

[36] Doets E.L., Ueland P.M., Tell G.S., Vollset S.E., Nygård O.K., Van’t Veer P., de Groot L.C., Nurk E., Refsum H., Smith A.D. (2014). Interactions between plasma concentrations of folate and markers of vitamin B(12) status with cognitive performance in elderly people not exposed to folic acid fortification: The Hordaland Health Study. Br. J. Nutr..

[37] Li F., Pei L., Huang G., Ye H. (2020). Influence of omega-3 fatty acid and vitamin co-supplementation on metabolic status in gestational diabetes: A meta-analysis of randomized controlled studies. Eur. J. Obstet. Gynecol. Reprod. Biol..

[38] Jamilian M., Mirhosseini N., Eslahi M., Bahmani F., Shokrpour M., Chamani M., Asemi Z. (2019). The effects of magnesium-zinc-calcium-vitamin D co-supplementation on biomarkers of inflammation, oxidative stress and pregnancy outcomes in gestational diabetes. BMC Pregnancy Childbirth.

[39] Zhang J., Bai S., Lin S., Du S., Zhao X., Qin Y., Yang X., Wang Z. (2024). The association between preterm birth and the supplementation with vitamin D and calcium during pregnancy. Clin. Nutr. ESPEN.

[40] Shukla V., Parvez S., Fatima G., Singh S., Magomedova A., Batiha G.E., Alexiou A., Papadakis M., Welson N.N., Hadi N. (2024). Micronutrient interactions: Magnesium and its synergies in maternal-fetal health. Food Sci. Nutr..

[41] Lu Q., Strodl E., Liang Y., Huang L.H., Hu B.J., Chen W.Q. (2023). Joint Effects of Prenatal Folic Acid Supplement with Prenatal Multivitamin and Iron Supplement on Obesity in Preschoolers Born SGA: Sex Specific Difference. Nutrients.

[42] Deng W., Wang H., Cai J. (2016). Analysis of developmental screening results using ASQ-3 among 2,246 infants aged 3 to 4 months in Shenzhen. J. Huazhong Univ. Sci. Technol. Med. Sci..

[43] Ruan Z.L., Liu L., Strodl E., Fan L.J., Yin X.N., Wen G.M., Sun D.L., Xian D.X., Jiang H., Jing J. (2017). Antenatal Training with Music and Maternal Talk Concurrently May Reduce Autistic-Like Behaviors at around 3 Years of Age. Front. Psychiatry.

[44] Fang X.Y., Strodl E., Liu B.Q., Liu L., Yin X.N., Wen G.M., Sun D.L., Xian D.X., Jiang H., Jing J. (2019). Association between prenatal exposure to household inhalants exposure and ADHD-like behaviors at around 3 years of age: Findings from Shenzhen Longhua Child Cohort Study. Environ. Res..

[45] Chen J., Strodl E., Huang L.H., Chen J.Y., Liu X.C., Yang J.H., Chen W.Q. (2021). Associations between Prenatal Education, Breastfeeding and Autistic-Like Behaviors in Pre-Schoolers. Children.

[46] Yang J., Gao L., Strodl E., Chen J., Tong F., Chen W. (2025). Impact of Breastfeeding Practices on Autistic Traits in Chinese Children Aged from 3 to 4 Years: Cross-Sectional Study. Nutrients.

[47] Wei M., Bian X., Squires J., Yao G., Wang X., Xie H., Song W., Lu J., Zhu C., Yue H. (2015). Studies of the norm and psychometrical properties of the ages and stages questionnaires, third edition, with a Chinese national sample. Chin. J. Pediatr..

[48] Agarwal P.K., Shi L., Daniel L.M., Yang P.H., Khoo P.C., Quek B.H., Zheng Q., Rajadurai V.S. (2017). Prospective evaluation of the Ages and Stages Questionnaire 3rd Edition in very-low-birthweight infants. Dev. Med. Child Neurol..

[49] Lopes S., Graça P., Teixeira S., Serrano A.M., Squires J. (2015). Psychometric properties and validation of Portuguese version of Ages & Stages Questionnaires (3rd edition): 9, 18 and 30 Questionnaires. Early Hum. Dev..

[50] Wang P., Xie J., Jiao X.C., Ma S.S., Liu Y., Yin W.J., Tao R.X., Hu H.L., Zhang Y., Chen X.X. (2021). Maternal Glycemia During Pregnancy and Early Offspring Development: A Prospective Birth Cohort Study. J. Clin. Endocrinol. Metab..

[51] Alving-Jessep E., Botchway E., Wood A.G., Hilton A.C., Blissett J.M. (2022). The development of the gut microbiome and temperament during infancy and early childhood: A systematic review. Dev. Psychobiol..

[52] Zhang D., Lan Y., Zhang J., Cao M., Yang X., Wang X. (2024). Effects of early-life gut microbiota on the neurodevelopmental outcomes of preterm infants: A multi-center, longitudinal observational study in China. Eur. J. Pediatr..

[53] Guo X., Xu J., Tian Y., Ouyang F., Yu X., Liu J., Yan C., Zhang J. (2024). Interaction of prenatal maternal selenium and manganese levels on child neurodevelopmental trajectories-the Shanghai birth cohort study. Sci. Total Environ..

[54] Allotey P.A., Harel O. (2019). Multiple Imputation for Incomplete Data in Environmental Epidemiology Research. Curr. Environ. Health Rep..

[55] Vecchione R., Westlake M., Bragg M.G., Rando J., Bennett D.H., Croen L.A., Dunlop A.L., Ferrara A., Hedderson M.M., Kerver J.M. (2024). Maternal Dietary Patterns During Pregnancy and Child Autism-Related Traits in the Environmental Influences on Child Health Outcomes Consortium. Nutrients.

[56] Tabaeifard R., Hashempour S., Karim Dehnavi M., Mofidi Nejad M., Omid N., Karimi M., Azadbakht L. (2025). Association between oxidative balance score and risk of postpartum depression in Iranian women: A prospective cohort study. Sci. Rep..

[57] Schottenfeld D. (2002). Epidemiology: An introduction. Am. J. Epidemiol..

[58] Knol M.J., VanderWeele T.J. (2012). Recommendations for presenting analyses of effect modification and interaction. Int. J. Epidemiol..

[59] Correia K., Williams P.L. (2018). Estimating the Relative Excess Risk Due to Interaction in Clustered-Data Settings. Am. J. Epidemiol..

[60] Bellocco R., Marrone G., Ye W., Nyrén O., Adami H.O., Mariosa D., Lagerros Y.T. (2016). A prospective cohort study of the combined effects of physical activity and anthropometric measures on the risk of post-menopausal breast cancer. Eur. J. Epidemiol..

[61] Zhu W., Zhu S., Sunguya B.F., Huang J. (2021). Urban–Rural Disparities in the Magnitude and Determinants of Stunting among Children under Five in Tanzania: Based on Tanzania Demographic and Health Surveys 1991–2016. Int. J. Environ. Res. Public Health.

[62] Zhu J., Xu P., Yan W., Hu Y., Guo H., Chen F., Bigambo F.M., Wang X. (2024). The Influence of Multivitamins on Neurological and Growth Disorders: A Cross-Sectional Study. Front. Nutr..

[63] Wei Q., Xiao Y., Yang T., Chen J., Chen L., Wang K., Zhang J., Li L., Jia F., Wu L. (2024). Predicting Autism Spectrum Disorder Using Maternal Risk Factors: A Multi-Center Machine Learning Study. Psychiatry Res..

[64] Cruz-Rodríguez J., Díaz-López A., Canals-Sans J., Arija V. (2023). Maternal Vitamin B_12_ Status during Pregnancy and Early Infant Neurodevelopment: The ECLIPSES Study. Nutrients.

[65] Rodgers M.D., Mead M.J., McWhorter C.A., Ebeling M.D., Shary J.R., Newton D.A., Baatz J.E., Gregoski M.J., Hollis B.W., Wagner C.L. (2023). Vitamin D and Child Neurodevelopment—A Post Hoc Analysis. Nutrients.

[66] Adams J.B., Kirby J.K., Sorensen J.C., Pollard E.L., Audhya T. (2022). Evidence Based Recommendations for an Optimal Prenatal Supplement for Women in the US: Vitamins and Related Nutrients. Matern. Health Neonatol. Perinatol..

[67] Ravikumar N., Chegukrishnamurthi M., Gadde Venkata S. (2022). Role of Micronutrients in Neurological Development. Role of Nutrients in Neurological Disorders.

[68] Guilarte T.R. (1993). Vitamin B_6_ and Cognitive Development: Recent Research Findings from Human and Animal Studies. Nutr. Rev..

[69] Benton D. (2012). Vitamins and Neural and Cognitive Developmental Outcomes in Children. Proc. Nutr. Soc..

[70] Tafti M., Ghyselinck N.B. (2007). Functional Implication of the Vitamin A Signaling Pathway in the Brain. Arch. Neurol..

[71] Chang J., Liu M., Liu C., Zhou S., Jiao Y., Sun H., Ji Y. (2024). Effects of Vitamins and Polyunsaturated Fatty Acids on Cognitive Function in Older Adults with Mild Cognitive Impairment: A Meta-Analysis of Randomized Controlled Trials. Eur. J. Nutr..

[72] Mantovani E., Filippini F., Bortolus R., Franchi M. (2014). Folic Acid Supplementation and Preterm Birth: Results from Observational Studies. BioMed Res. Int..

[73] WHO Periconceptional Folic Acid Supplementation to Prevent Neural Tube Defects. https://www.who.int/tools/elena/interventions/folate-periconceptional.

[74] WHO WHO Model List of Essential Medicines 19th Edition. https://publichealthupdate.com/who-model-list-of-essential-medicines-april-2015-19th-edition/.

[75] Chmielewska A., Dziechciarz P., Gieruszczak-Białek D., Horvath A., Pieścik-Lech M., Ruszczyński M., Skórka A., Szajewska H. (2019). Effects of Prenatal and/or Postnatal Supplementation with Iron, PUFA or Folic Acid on Neurodevelopment: Update. Br. J. Nutr..

[76] Caffrey A., McNulty H., Rollins M., Prasad G., Gaur P., Talcott J.B., Witton C., Cassidy T., Marshall B., Dornan J. (2021). Effects of Maternal Folic Acid Supplementation during the Second and Third Trimesters of Pregnancy on Neurocognitive Development in the Child: An 11-Year Follow-Up from a Randomised Controlled Trial. BMC Med..

[77] Raghavan R., Riley A.W., Volk H., Caruso D., Hironaka L., Sices L., Hong X., Wang G., Ji Y., Brucato M. (2018). Maternal Multivitamin Intake, Plasma Folate and Vitamin B_12_ Levels and Autism Spectrum Disorder Risk in Offspring. Paediatr. Perinat. Epidemiol..

[78] McGowan E.C., Hong X., Selhub J., Paul L., Wood R.A., Matsui E.C., Keet C.A., Wang X. (2020). Association Between Folate Metabolites and the Development of Food Allergy in Children. J. Allergy Clin. Immunol. Pract..

[79] Díaz-Piña D.A., Rivera-Ramírez N., García-López G., Díaz N.F., Molina-Hernández A. (2024). Calcium and Neural Stem Cell Proliferation. Int. J. Mol. Sci..

[80] Prado E.L., Dewey K.G. (2014). Nutrition and Brain Development in Early Life. Nutr. Rev..

[81] Kiely M.E., McCarthy E.K., Hennessy Á. (2021). Iron, Iodine and Vitamin D Deficiencies during Pregnancy: Epidemiology, Risk Factors and Developmental Impacts. Proc. Nutr. Soc..

[82] Rodionov D.A., Arzamasov A.A., Khoroshkin M.S., Iablokov S.N., Leyn S.A., Peterson S.N., Novichkov P.S., Osterman A.L. (2019). Micronutrient Requirements and Sharing Capabilities of the Human Gut Microbiome. Front. Microbiol..

[83] Berridge M.J. (2018). Vitamin D Deficiency: Infertility and Neurodevelopmental Diseases (Attention Deficit Hyperactivity Disorder, Autism, and Schizophrenia). Am. J. Physiol. Cell Physiol..

[84] Gusso D., Prauchner G.R.K., Rieder A.S., Wyse A.T.S. (2023). Biological Pathways Associated with Vitamins in Autism Spectrum Disorder. Neurotox. Res..

[85] Hill T.R., Verlaan S., Biesheuvel E., Eastell R., Bauer J.M., Bautmans I., Brandt K., Donini L.M., Maggio M., Mets T. (2019). A Vitamin D, Calcium and Leucine-Enriched Whey Protein Nutritional Supplement Improves Measures of Bone Health in Sarcopenic Non-Malnourished Older Adults: The PROVIDE Study. Calcif. Tissue Int..

[86] Gariballa S., Yasin J., Alessa A. (2022). A Randomized, Double-Blind, Placebo-Controlled Trial of Vitamin D Supplementation with or without Calcium in Community-Dwelling Vitamin D Deficient Subjects. BMC Musculoskelet. Disord..

[87] Liu S.H., Bobb J.F., Claus Henn B., Gennings C., Schnaas L., Tellez-Rojo M., Bellinger D., Arora M., Wright R.O., Coull B.A. (2018). Bayesian Varying Coefficient Kernel Machine Regression to Assess Neurodevelopmental Trajectories Associated with Exposure to Complex Mixtures. Stat. Med..

[88] Zhong C., Tessing J., Lee B.K., Lyall K. (2020). Maternal Dietary Factors and the Risk of Autism Spectrum Disorders: A Systematic Review of Existing Evidence. Autism Res. Off. J. Int. Soc. Autism Res..

[89] Li N., Zhao G., Wu W., Zhang M., Liu W., Chen Q., Wang X. (2020). The Efficacy and Safety of Vitamin C for Iron Supplementation in Adult Patients with Iron Deficiency Anemia: A Randomized Clinical Trial. JAMA Netw. Open.

[90] Rocha Dda S., Capanema F.D., Netto M.P., de Almeida C.A., Franceschini Sdo C., Lamounier J.A. (2011). Effectiveness of Fortification of Drinking Water with Iron and Vitamin C in the Reduction of Anemia and Improvement of Nutritional Status in Children Attending Day-Care Centers in Belo Horizonte, Brazil. Food Nutr. Bull..

[91] da Cunha M.S., Siqueira E.M., Trindade L.S., Arruda S.F. (2014). Vitamin A Deficiency Modulates Iron Metabolism via Ineffective Erythropoiesis. J. Nutr. Biochem..

[92] Kennedy D.O. (2016). B Vitamins and the Brain: Mechanisms, Dose and Efficacy—A Review. Nutrients.

[93] Ahangari R., Mohammadbeigi A., Miraj S., Rajabi N., Mohammadpour R. (2024). Monitoring the Utilization and Effectiveness of Iron and Vitamin D Supplementations Program and Its Predictive Factors in High Schools’ Girls in Qom, Iran. J. Prev. Med. Hyg..

[94] Mukherjee R., Gupta Bansal P., Lyngdoh T., Medhi B., Sharma K.A., Prashanth T., Pullakhandam R., Chowdhury R., Taneja S., Yadav K. (2024). Recommendations for India-Specific Multiple Micronutrient Supplement Through Expert Consultation. Indian J. Med Res..

[95] Bourre J.M. (2006). Effects of Nutrients (in Food) on the Structure and Function of the Nervous System: Update on Dietary Requirements for Brain. Part 1: Micronutrients. J. Nutr. Health Aging.

[96] Moerkerke M., Daniels N., Van der Donck S., Tang T., Prinsen J., Yargholi E., Steyaert J., Alaerts K., Boets B. (2024). Impact of Chronic Intranasal Oxytocin Administration on Face Expression Processing in Autistic Children: A Randomized Controlled Trial Using fMRI. Mol. Autism.

