# Supplementary material for: The Individual and Combined Effects of Prenatal Micronutrient Supplementations on Neurobehavioral Developmental Disorders in Preschool Children
Source: Children (Basel). 2025 May 5;12(5):602. doi: 10.3390/children12050602 (PMC12110273; doi:10.3390/children12050602)
Supplement: Supplementary file 1 [file children-12-00602-s001.zip › Table S1_definition&code.pdf]

Supplementary Table 1 Definitions and coding used in the questionnaire of 2022 children’s survey.

Variable	Question	Values
Child’s age	What is the child's birth date?	-
Birth weight	What was the birth weight of child? (kg)	-
Maternal/ paternal age of conception	What is the mother's birth date?	Mother's age - child's age
	What is the father's birth date?	Father's age - child's age
Child’s sex	What is the sex of the child?	1= Male
		2= Female
Birth season	What is the child's birth date?	1= Spring
		2= Summer
		3= Autumn
		4= Winter

Residence type	What is the child's residence type?	1 = Shenzhen residents<br>2 = Non-Shenzhen residents (including temporary and floating residents)
Separation from mother/father	Was the child ever separated from mother/father for more than 6 months?	1= No<br>2= Yes
Marital status	What is the mother's marital status?	1= Not married (including single, divorced and widowed)<br>2= Married (including married and remarried)
Mother/father education	What is the mother's/father’s education level?	1= Less than high school<br>2= High school and higher
Household income	What is your total monthly household income?	1= <RMB 20,000<br>2= ≥RMB 20,000

Pregnancy mode	What was the pregnancy mode for this child?	1= Natural pregnancy<br>2= Medically assisted pregnancy
Threatened abortion (TA)	Did the mother have a threatened abortion during the pregnancy?	1= No<br>2= Yes
Intrauterine growth retardation (IUGR)	Was the child diagnosed with intrauterine growth retardation?	1= No<br>2= Yes
Congenital diseases	Was the child diagnosed with congenital diseases?	1= No<br>2= Yes
Pregestational diabetes mellitus (PGDM)	Had the mother ever been diagnosed with diabetes mellitus before pregnancy with this child?	1= No<br>2= Yes
Pregestational hypertension(PGH)	Had the mother ever been diagnosed with hypertension before pregnancy with this child?	1= No<br>2= Yes

Pregestational mental disease(PGMD)	Had the mother ever been diagnosed with mental disease before pregnancy with this child?	1= No<br>2= Yes
Polycystic ovarian syndrome (POS)	Had the mother ever been diagnosed with polycystic ovarian syndrome before pregnancy with this child?	1= No<br>2= Yes
Gestational hypertension (GH)	Was the mother diagnosed with gestational hypertension during the pregnancy with this child?	1= No<br>2= Yes
Pre-eclampsia (PE)	Was the mother diagnosed with pre-eclampsia during the pregnancy with this child?	1= No<br>2= Yes
Gestational diabetes mellitus (GDM)	Was the mother diagnosed with gestational diabetes mellitus during the pregnancy with this	1= No<br>2= Yes

	child?	
Pregnancy Chinese medicine intake	Did the mother take Chinese medicine during the pregnancy?	1= No<br>2= Yes
Pregnancy preservation	Did the mother take pregnancy preservation during the pregnancy?	1= No<br>2= Yes
Pregnancy seminar participation	Did the mother participate in pregnancy seminars during the pregnancy?	1= No<br>2= Yes
Pre-pregnancy BMI	What were the mother's height (m) and weight (kg) before this pregnancy?	1= BMI <18.5 kg/m^2<br>2= BMI 18.5-23.9 kg/m^2<br>3= BMI ≥24 kg/m^2
Parity	How many times had the mother given birth before this child?	1= Nulliparous<br>2= Multiparous

Multiple pregnancy	How many fetuses was the mother carrying?	1 = Single pregnancy<br>2 = Multiple pregnancy
Delivery mode	What was the mother's delivery model for this child?	1= Natural birth<br>2= Cesarean<br>3= Vaginal surgery for delivery
Preterm birth (PTB)	What was the gestational age of child? (Week)	1 = No (≥37 weeks)<br>2 = Yes (<37 weeks)
Feeding pattern	What was the child's feeding pattern in early childhood?	1= Breastfeeding<br>2= Formula feeding<br>3= Mixed feeding
Child’s nutritional condition	What was the child's nutritional status in early childhood?	1= Bad (very bad/ bad)<br>2= Good (general/good/very good)

Child’s health condition	What was the child's health status in early childhood?	1= Bad (very bad/ bad)<br>2= Good (general/good/very good)
Mother/father smoking	Does the mother/father smoke?	1= No<br>2= Yes
Mother/father drinking	Does the mother/father drink alcohol?	1= No<br>2= Yes
Parental depression	PHQ-9	1= No (Score >4)<br>2= Yes (Score ≤4)
Family function	Family APGAR scale	1= Normal (Score ≥7)<br>2= Dysfunction (Score <7)
