# Supplementary material for: The Individual and Combined Effects of Prenatal Micronutrient Supplementations on Neurobehavioral Developmental Disorders in Preschool Children
Source: Children (Basel). 2025 May 5;12(5):602. doi: 10.3390/children12050602 (PMC12110273; doi:10.3390/children12050602)
Supplement: Supplementary file 1 [file children-12-00602-s001.zip › Table S3 individual effect.pdf]

Supplementary Table 3 Individual effects of micronutrients on NDDs in the crude, adjusted and full-inclusion model.

Variables	NDDs						
		Crude Model		Adjusted Model[a]		Full-inclusion Model[b]	
	N(%)	OR(95%CI)	*p*-value	OR(95%CI)	*p-value*	OR(95%CI)	*p-value*
Calcium							
No	495 (13.1)	1.00		1.00		1.00	
Yes	1,337 (11.3)	0.84 (0.76,0.94)	**0.003**	0.92 (0.82,1.03)	0.16	0.93 (0.80,1.07)	0.32
Folic acid							
No	249 (13.5)	1.00		1.00		1.00	
Yes	1,583 (11.5)	0.83 (0.72,0.96)	**0.01**	0.90 (0.78,1.05)	0.18	0.93 (0.78,1.11)	0.42
Iron							
No	1,023 (12.1)	1.00		1.00		1.00	
Yes	809 (11.3)	0.92 (0.84,1.02)	0.10	1.05 (0.95,1.17)	0.30	1.14 (1.01,1.28)	0.03
Multivitamin							
No	1,141 (13.1)	1.00		1.00		1.00	
Yes	691 (9.9)	0.73 (0.66,0.81)	**<0.001**	0.86 (0.78,0.96)	**0.007**	0.85 (0.75,0.95)	**0.006**

[a] Adjusted Model: Adjusted for child's basic characteristics, maternal demographic characteristics, pregnancy and perinatal characteristics and childhood family environment.

[b] Full-inclusion Model: Included all micronutrients in the model and adjusted for child's basic characteristics, maternal demographic characteristics, pregnancy and perinatal characteristics and childhood family environment.
