# Supplementary material for: The Individual and Combined Effects of Prenatal Micronutrient Supplementations on Neurobehavioral Developmental Disorders in Preschool Children
Source: Children (Basel). 2025 May 5;12(5):602. doi: 10.3390/children12050602 (PMC12110273; doi:10.3390/children12050602)
Supplement: Supplementary file 1 [file children-12-00602-s001.zip › Table S5 combined effect_5 domain.pdf]

Supplementary Table 5 Combined effects of micronutrients on NDDs across five domains in the crude, adjusted and full-inclusion model.

Micronutrients		Crude model					Adjusted model [a]				Full-inclusion model [b]			
		N (%)	OR	IOR	RERI	AP	OR	IOR	RERI	AP	OR	IOR	RERI	AP
**Total**														
Calcium	Folic acid													
No	No	210 (13.5%)	1.00 (ref)				1.00 (ref)				1.00 (ref)			
No	Yes	39 (13.3%)	0.98 (0.67, 1.40)				1.00 (0.68, 1.43)				0.99 (0.67, 1.43)			
Yes	No	285 (12.8%)	0.94 (0.77, 1.14)				0.95 (0.78, 1.16)				0.96 (0.79, 1.17)			
Yes	Yes	1298 (11.2%)	**0.81 (0.69, 0.95)**	0.88 (0.60, 1.32)	-0.11 (-0.49, 0.28)	-0.13 (-0.60, 0.34)	0.89 (0.76, 1.05)	0.94 (0.64, 1.42)	-0.05 (-0.45, 0.35)	-0.06 (-0.51, 0.38)	0.89 (0.75, 1.06)	0.94 (0.63, 1.41)	-0.06 (-0.46, 0.34)	-0.07 (-0.51, 0.38)
Calcium	Iron													
No	No	457 (13.2%)	1.00 (ref)				1.00 (ref)				1.00 (ref)			
No	Yes	38 (12.3%)	0.93 (0.64, 1.30)				0.98 (0.67, 1.39)				1.02 (0.69, 1.45)			
Yes	No	566 (11.4%)	**0.85 (0.74, 0.97)**				**0.86 (0.76, 0.99)**				0.91 (0.78, 1.07)			
Yes	Yes	771 (11.2%)	**0.83 (0.74, 0.94)**	1.06 (0.74, 1.56)	0.06 (-0.28, 0.40)	0.07 (-0.34, 0.49)	0.97 (0.85, 1.10)	1.15 (0.79, 1.70)	0.13 (-0.24, 0.49)	0.13 (-0.25, 0.51)	1.06 (0.91, 1.24)	1.14 (0.79, 1.70)	0.13 (-0.25, 0.51)	0.12 (-0.24, 0.49)
Calcium	Multivitamin													
No	No	423 (13.3%)	1.00 (ref)				1.00 (ref)				1.00 (ref)			
No	Yes	718 (13%)	0.98 (0.86, 1.12)				1.01 (0.89, 1.16)				1.00 (0.85, 1.17)			
Yes	No	72 (12.1%)	0.90 (0.68, 1.17)				1.08 (0.82, 1.42)				1.10 (0.83, 1.45)			
Yes	Yes	619 (9.7%)	**0.71 (0.62, 0.80)**	0.80 (0.60, 1.08)	-0.17 (-0.44, 0.10)	-0.25 (-0.62, 0.13)	**0.85 (0.74, 0.98)**	0.78 (0.58, 1.05)	-0.24 (-0.56, 0.08)	-0.28 (-0.65, 0.09)	**0.82 (0.69, 0.97)**	0.74 (0.55, 1.01)	-0.28 (-0.62, 0.05)	-0.35 (-0.75, 0.05)
Folic acid	Iron													
No	No	233 (13.8%)	1.00 (ref)				1.00 (ref)				1.00 (ref)			
No	Yes	16 (10.5%)	0.74 (0.42, 1.22)				0.80 (0.45, 1.34)				0.87 (0.48, 1.47)			
Yes	No	790 (11.7%)	**0.83 (0.71, 0.97)**				**0.85 (0.73, 1.00)**				0.92 (0.76, 1.10)			
Yes	Yes	793 (11.3%)	**0.80 (0.68, 0.93)**	1.30 (0.78, 2.33)	0.23 (-0.18, 0.63)	0.29 (-0.23, 0.81)	0.93 (0.79, 1.10)	1.37 (0.81, 2.46)	0.28 (-0.16, 0.72)	0.30 (-0.19, 0.78)	1.06 (0.86, 1.30)	1.33 (0.78, 2.41)	0.27 (-0.21, 0.76)	0.26 (-0.21, 0.72)

Micronutrients		Crude model					Adjusted model [a]				Full-inclusion model [b]			
		N (%)	OR	IOR	RERI	AP	OR	IOR	RERI	AP	OR	IOR	RERI	AP
Folic acid	Multivitamin													
No	No	235 (14.1%)	1.00 (ref)				1.00 (ref)				1.00 (ref)			
No	Yes	906 (12.9%)	0.91 (0.78, 1.06)				0.91 (0.78, 1.07)				0.91 (0.76, 1.10)			
Yes	No	14 (8.0%)	**0.53 (0.29, 0.90)**				0.60 (0.33, 1.03)				0.60 (0.33, 1.04)			
Yes	Yes	677 (10%)	**0.68 (0.58, 0.80)**	1.41 (0.82, 2.61)	0.24 (-0.08, 0.56)	0.36 (-0.13, 0.84)	**0.81 (0.69, 0.96)**	1.47 (0.85, 2.74)	0.29 (-0.07, 0.65)	0.36 (-0.09, 0.82)	**0.79 (0.65, 0.97)**	1.43 (0.83, 2.68)	0.27 (-0.09, 0.64)	0.35 (-0.13, 0.82)
Iron	Multivitamin													
No	No	795 (13.4%)	1.00 (ref)				1.00 (ref)				1.00 (ref)			
No	Yes	346 (12.6%)	0.93 (0.81, 1.06)				1.03 (0.89, 1.18)				1.06 (0.91, 1.23)			
Yes	No	228 (9.1%)	**0.64 (0.55, 0.75)**				**0.76 (0.65, 0.89)**				**0.78 (0.66, 0.92)**			
Yes	Yes	463 (10.5%)	**0.75 (0.67, 0.85)**	**1.26 (1.02, 1.57)**	**0.18 (0.02, 0.35)**	0.24 (0.02, 0.46)	0.94 (0.83, 1.07)	1.21 (0.98, 1.51)	0.16 (-0.04, 0.35)	0.17 (-0.04, 0.37)	0.98 (0.85, 1.13)	1.18 (0.95, 1.48)	0.14 (-0.06, 0.34)	0.14 (-0.06, 0.35)
**Communication domain**														
Calcium	Folic acid													
No	No	28 (1.8%)	1 (ref)				1 (ref)				1 (ref)			
No	Yes	4 (1.4%)	0.75 (0.22, 1.93)				0.76 (0.22, 1.99)				0.83 (0.24, 2.18)			
Yes	No	31 (1.4%)	0.77 (0.46, 1.29)				0.75 (0.45, 1.27)				0.78 (0.46, 1.32)			
Yes	Yes	116 (1%)	**0.55 (0.36, 0.84)**	0.96 (0.34, 3.44)	0.03 (-0.84, 0.91)	0.06 (-1.53, 1.65)	**0.61 (0.39, 0.93)**	1.05 (0.37, 3.81)	0.09 (-0.79, 0.97)	0.15 (-1.33, 1.63)	0.68 (0.42, 1.08)	1.05 (0.37, 3.79)	0.07 (-0.88, 1.02)	0.1 (-1.31, 1.51)
Calcium	Iron													
No	No	57 (1.6%)	1 (ref)				1 (ref)				1 (ref)			
No	Yes	2 (0.6%)	0.39 (0.06, 1.26)				0.4 (0.07, 1.31)				0.45 (0.07, 1.49)			
Yes	No	56 (1.1%)	**0.68 (0.47, 0.99)**				0.7 (0.48, 1.02)				0.79 (0.52, 1.23)			
Yes	Yes	64 (0.9%)	**0.56 (0.39, 0.81)**	2.12 (0.62, 13.32)	0.49 (-0.11, 1.09)	0.87 (-0.24, 1.99)	**0.67 (0.46, 0.96)**	2.36 (0.68, 14.87)	0.56 (-0.06, 1.19)	0.85 (-0.13, 1.82)	0.79 (0.51, 1.23)	2.22 (0.63, 14.1)	0.55 (-0.16, 1.25)	0.69 (-0.25, 1.63)
Calcium	Multivitamin													
No	No	50 (1.6%)	1 (ref)				1 (ref)				1 (ref)			

Micronutrients		Crude model					Adjusted model [a]				Full-inclusion model [b]			
		N (%)	OR	IOR	RERI	AP	OR	IOR	RERI	AP	OR	IOR	RERI	AP
No	Yes	70 (1.3%)	0.81 (0.56, 1.17)				0.83 (0.58, 1.21)				0.97 (0.62, 1.52)			
Yes	No	9 (1.5%)	0.96 (0.44, 1.87)				1.11 (0.5, 2.2)				1.24 (0.55, 2.52)			
Yes	Yes	50 (0.8%)	**0.5 (0.34, 0.74)**	0.64 (0.3, 1.51)	-0.27 (-1.04, 0.49)	-0.55 (-2.06, 0.96)	**0.61 (0.41, 0.92)**	0.66 (0.31, 1.57)	-0.33 (-1.2, 0.54)	-0.54 (-1.93, 0.86)	0.73 (0.44, 1.2)	0.6 (0.27, 1.45)	-0.49 (-1.5, 0.53)	-0.67 (-2.02, 0.68)
Folic acid	Iron													
No	No	31 (1.8%)	1 (ref)				1 (ref)				1 (ref)			
No	Yes	1 (0.7%)	0.36 (0.02, 1.67)				0.38 (0.02, 1.82)				0.44 (0.02, 2.17)			
Yes	No	82 (1.2%)	0.66 (0.44, 1.01)				0.66 (0.44, 1.02)				0.74 (0.46, 1.22)			
Yes	Yes	65 (0.9%)	**0.5 (0.33, 0.77)**	2.14 (0.44, 38.68)	0.49 (-0.26, 1.23)	0.97 (-0.62, 2.56)	**0.58 (0.37, 0.91)**	2.31 (0.47, 41.88)	0.54 (-0.26, 1.33)	0.93 (-0.53, 2.39)	0.71 (0.4, 1.26)	2.17 (0.43, 39.57)	0.53 (-0.39, 1.44)	0.74 (-0.65, 2.14)
Folic acid	Multivitamin													
No	No	30 (1.8%)	1 (ref)				1 (ref)				1 (ref)			
No	Yes	90 (1.3%)	0.71 (0.47, 1.09)				0.7 (0.47, 1.09)				0.78 (0.48, 1.28)			
Yes	No	2 (1.1%)	0.63 (0.1, 2.12)				0.72 (0.11, 2.43)				0.78 (0.12, 2.71)			
Yes	Yes	57 (0.8%)	**0.46 (0.3, 0.72)**	1.03 (0.29, 6.58)	0.12 (-0.83, 1.08)	0.26 (-1.82, 2.35)	**0.55 (0.35, 0.88)**	1.1 (0.31, 7.04)	0.13 (-0.94, 1.21)	0.24 (-1.72, 2.2)	0.64 (0.37, 1.13)	1.05 (0.29, 6.75)	0.08 (-1.1, 1.26)	0.12 (-1.73, 1.97)
Iron	Multivitamin													
No	No	89 (1.5%)	1 (ref)				1 (ref)				1 (ref)			
No	Yes	31 (1.1%)	0.75 (0.49, 1.11)				0.84 (0.54, 1.26)				0.94 (0.59, 1.47)			
Yes	No	24 (1%)	**0.63 (0.39, 0.98)**				0.75 (0.46, 1.18)				0.83 (0.5, 1.33)			
Yes	Yes	35 (0.8%)	**0.52 (0.35, 0.77)**	1.11 (0.57, 2.18)	0.14 (-0.3, 0.59)	0.27 (-0.57, 1.12)	**0.67 (0.44, 1)**	1.06 (0.55, 2.08)	0.08 (-0.44, 0.6)	0.11 (-0.66, 0.89)	0.76 (0.48, 1.2)	0.97 (0.5, 1.93)	-0.01 (-0.61, 0.58)	-0.02 (-0.8, 0.76)
							**Gross motor domain**							
Calcium	Folic acid													
No	No	166 (10.7%)	1 (ref)				1 (ref)				1 (ref)			
No	Yes	31 (10.5%)	0.98 (0.65, 1.46)				1.02 (0.66, 1.53)				1.02 (0.66, 1.54)			
Yes	No	216 (9.7%)	0.9 (0.72, 1.11)				0.93 (0.75, 1.16)				0.94 (0.76, 1.17)			

Micronutrients		Crude model					Adjusted model [a]				Full-inclusion model [b]			
		N (%)	OR	IOR	RERI	AP	OR	IOR	RERI	AP	OR	IOR	RERI	AP
Yes	Yes	975 (8.4%)	**0.77 (0.65, 0.92)**	0.87 (0.57, 1.37)	-0.11 (-0.54, 0.32)	-0.14 (-0.69, 0.41)	0.86 (0.72, 1.03)	0.91 (0.59, 1.44)	-0.09 (-0.54, 0.36)	-0.1 (-0.62, 0.42)	0.87 (0.72, 1.06)	0.9 (0.59, 1.43)	-0.09 (-0.55, 0.36)	-0.11 (-0.62, 0.41)
Calcium	Iron													
No	No	349 (10.1%)	1 (ref)				1 (ref)				1 (ref)			
No	Yes	33 (10.7%)	1.07 (0.72, 1.54)				1.15 (0.77, 1.67)				1.22 (0.81, 1.78)			
Yes	No	439 (8.8%)	**0.87 (0.75, 1)**				0.89 (0.76, 1.03)				0.95 (0.8, 1.14)			
Yes	Yes	567 (8.2%)	**0.8 (0.7, 0.93)**	0.87 (0.59, 1.31)	-0.13 (-0.55, 0.29)	-0.16 (-0.68, 0.36)	0.94 (0.81, 1.09)	0.92 (0.62, 1.41)	-0.09 (-0.55, 0.36)	-0.1 (-0.58, 0.39)	1.05 (0.88, 1.25)	0.9 (0.6, 1.39)	-0.12 (-0.61, 0.37)	-0.12 (-0.58, 0.35)
Calcium	Multivitamin													
No	No	329 (10.3%)	1 (ref)				1 (ref)				1 (ref)			
No	Yes	545 (9.9%)	0.95 (0.83, 1.1)				0.99 (0.85, 1.14)				0.99 (0.83, 1.19)			
Yes	No	53 (8.9%)	0.85 (0.62, 1.14)				1.06 (0.77, 1.43)				1.09 (0.79, 1.49)			
Yes	Yes	461 (7.3%)	**0.68 (0.59, 0.79)**	0.84 (0.61, 1.18)	-0.12 (-0.42, 0.17)	-0.18 (-0.61, 0.24)	**0.84 (0.72, 0.98)**	0.8 (0.58, 1.13)	-0.21 (-0.56, 0.15)	-0.25 (-0.67, 0.17)	**0.83 (0.68, 1)**	0.76 (0.54, 1.08)	-0.26 (-0.64, 0.12)	-0.32 (-0.76, 0.13)
Folic acid	Iron													
No	No	183 (10.8%)	1 (ref)				1 (ref)				1 (ref)			
No	Yes	14 (9.2%)	0.84 (0.45, 1.43)				0.94 (0.51, 1.63)				1.02 (0.54, 1.78)			
Yes	No	605 (9%)	**0.81 (0.68, 0.97)**				0.85 (0.71, 1.01)				0.91 (0.74, 1.12)			
Yes	Yes	586 (8.3%)	**0.75 (0.63, 0.89)**	1.1 (0.64, 2.06)	0.1 (-0.39, 0.59)	0.13 (-0.52, 0.79)	0.89 (0.75, 1.07)	1.12 (0.64, 2.11)	0.11 (-0.45, 0.66)	0.12 (-0.5, 0.74)	1.01 (0.8, 1.28)	1.09 (0.62, 2.07)	0.09 (-0.52, 0.69)	0.08 (-0.51, 0.68)
Folic acid	Multivitamin													
No	No	186 (11.1%)	1 (ref)				1 (ref)				1 (ref)			
No	Yes	688 (9.8%)	0.87 (0.73, 1.03)				0.89 (0.74, 1.06)				0.89 (0.73, 1.09)			
Yes	No	11 (6.3%)	**0.54 (0.27, 0.96)**				0.63 (0.31, 1.14)				0.63 (0.32, 1.15)			
Yes	Yes	503 (7.4%)	**0.64 (0.54, 0.77)**	1.38 (0.76, 2.77)	0.24 (-0.12, 0.6)	0.37 (-0.21, 0.95)	**0.79 (0.65, 0.95)**	1.41 (0.77, 2.85)	0.27 (-0.15, 0.69)	0.35 (-0.2, 0.89)	**0.78 (0.62, 0.97)**	1.38 (0.75, 2.79)	0.25 (-0.17, 0.68)	0.33 (-0.23, 0.89)

Micronutrients		Crude model					Adjusted model [a]				Full-inclusion model [b]			
		N (%)	OR	IOR	RERI	AP	OR	IOR	RERI	AP	OR	IOR	RERI	AP
Iron	Multivitamin													
No	No	615 (10.4%)	1 (ref)				1 (ref)				1 (ref)			
No	Yes	259 (9.4%)	0.9 (0.77, 1.04)				1 (0.85, 1.17)				1.04 (0.88, 1.23)			
Yes	No	173 (6.9%)	**0.64 (0.53, 0.76)**				**0.77 (0.64, 0.92)**				**0.79 (0.66, 0.96)**			
Yes	Yes	341 (7.7%)	**0.72 (0.63, 0.83)**	1.26 (0.99, 1.61)	**0.19 (0, 0.37)**	0.26 (0, 0.51)	0.92 (0.79, 1.06)	1.2 (0.94, 1.54)	0.15 (-0.06, 0.37)	0.17 (-0.07, 0.4)	0.96 (0.82, 1.13)	1.17 (0.91, 1.5)	0.13 (-0.1, 0.36)	0.13 (-0.1, 0.37)
**Fine motor domain**														
Calcium	Folic acid													
No	No	38 (2.4%)	1 (ref)				1 (ref)				1 (ref)			
No	Yes	6 (2%)	0.83 (0.31, 1.84)				0.83 (0.31, 1.85)				0.81 (0.3, 1.82)			
Yes	No	59 (2.6%)	1.08 (0.72, 1.65)				1.06 (0.7, 1.63)				1.07 (0.7, 1.63)			
Yes	Yes	319 (2.8%)	1.13 (0.8, 1.59)	1.26 (0.54, 3.46)	0.22 (-0.56, 1)	0.19 (-0.52, 0.9)	1.25 (0.88, 1.77)	1.43 (0.61, 3.96)	0.36 (-0.41, 1.14)	0.29 (-0.35, 0.93)	1.22 (0.84, 1.77)	1.42 (0.6, 3.94)	0.35 (-0.41, 1.11)	0.29 (-0.36, 0.93)
Calcium	Iron													
No	No	89 (2.6%)	1 (ref)				1 (ref)				1 (ref)			
No	Yes	8 (2.6%)	1.01 (0.45, 1.98)				1.04 (0.46, 2.06)				1.01 (0.44, 2.02)			
Yes	No	136 (2.7%)	1.07 (0.82, 1.4)				1.11 (0.84, 1.47)				1.08 (0.79, 1.47)			
Yes	Yes	189 (2.7%)	1.07 (0.83, 1.39)	1 (0.49, 2.31)	0 (-0.78, 0.77)	0 (-0.73, 0.72)	1.28 (0.98, 1.67)	1.11 (0.54, 2.59)	0.13 (-0.68, 0.94)	0.1 (-0.53, 0.73)	1.27 (0.93, 1.74)	1.17 (0.56, 2.77)	0.19 (-0.6, 0.98)	0.15 (-0.48, 0.77)
Calcium	Multivitamin													
No	No	81 (2.5%)	1 (ref)				1 (ref)				1 (ref)			
No	Yes	174 (3.2%)	1.25 (0.96, 1.64)				1.31 (1.01, 1.73)				1.19 (0.87, 1.64)			
Yes	No	16 (2.7%)	1.06 (0.59, 1.77)				1.23 (0.68, 2.09)				1.18 (0.65, 2.02)			
Yes	Yes	151 (2.4%)	0.93 (0.71, 1.23)	0.7 (0.4, 1.31)	-0.38 (-1.03, 0.28)	-0.4 (-1.09, 0.28)	1.15 (0.86, 1.53)	0.71 (0.4, 1.32)	-0.4 (-1.15, 0.35)	-0.35 (-0.98, 0.29)	1 (0.71, 1.41)	0.72 (0.4, 1.35)	-0.36 (-1.09, 0.37)	-0.36 (-1.06, 0.34)
Folic acid	Iron													
No	No	39 (2.3%)	1 (ref)				1 (ref)				1 (ref)			
No	Yes	5 (3.3%)	1.44 (0.49, 3.4)				1.52 (0.51, 3.64)				1.47 (0.49, 3.61)			

Micronutrients		Crude model					Adjusted model [a]				Full-inclusion model [b]			
		N (%)	OR	IOR	RERI	AP	OR	IOR	RERI	AP	OR	IOR	RERI	AP
Yes	No	186 (2.8%)	1.2 (0.86, 1.73)				1.22 (0.87, 1.77)				1.19 (0.81, 1.79)			
Yes	Yes	192 (2.7%)	1.19 (0.84, 1.69)	0.69 (0.28, 2.05)	-0.45 (-1.84, 0.93)	-0.38 (-1.52, 0.76)	1.4 (0.98, 2.01)	0.75 (0.31, 2.28)	-0.34 (-1.8, 1.13)	-0.24 (-1.27, 0.79)	1.38 (0.89, 2.14)	0.79 (0.32, 2.4)	-0.28 (-1.7, 1.15)	-0.2 (-1.22, 0.81)
Folic acid	Multivitamin													
No	No	39 (2.3%)	1 (ref)				1 (ref)				1 (ref)			
No	Yes	216 (3.1%)	1.33 (0.95, 1.9)				1.34 (0.95, 1.93)				1.21 (0.83, 1.81)			
Yes	No	5 (2.9%)	1.23 (0.42, 2.89)				1.38 (0.47, 3.28)				1.27 (0.43, 3.06)			
Yes	Yes	162 (2.4%)	1.03 (0.72, 1.46)	0.63 (0.26, 1.87)	-0.53 (-1.76, 0.69)	-0.52 (-1.67, 0.63)	1.23 (0.85, 1.77)	0.66 (0.27, 2)	-0.49 (-1.85, 0.86)	-0.4 (-1.48, 0.68)	1.05 (0.68, 1.61)	0.68 (0.28, 2.06)	-0.43 (-1.69, 0.83)	-0.41 (-1.57, 0.75)
Iron	Multivitamin													
No	No	173 (2.9%)	1 (ref)				1 (ref)				1 (ref)			
No	Yes	82 (3%)	1.02 (0.78, 1.33)				1.14 (0.86, 1.49)				1.06 (0.79, 1.41)			
Yes	No	52 (2.1%)	**0.7 (0.51, 0.95)**				0.82 (0.59, 1.13)				0.77 (0.55, 1.07)			
Yes	Yes	115 (2.6%)	0.89 (0.7, 1.13)	1.24 (0.81, 1.9)	0.16 (-0.2, 0.52)	0.18 (-0.22, 0.59)	1.12 (0.87, 1.45)	1.2 (0.78, 1.85)	0.16 (-0.26, 0.58)	0.14 (-0.23, 0.51)	1.03 (0.78, 1.36)	1.26 (0.82, 1.96)	0.2 (-0.19, 0.6)	0.2 (-0.19, 0.58)
**Problem solving domain**														
Calcium	Folic acid													
No	No	19 (1.2%)	1 (ref)				1 (ref)				1 (ref)			
No	Yes	3 (1%)	0.83 (0.19, 2.46)				0.9 (0.21, 2.7)				0.96 (0.22, 2.91)			
Yes	No	18 (0.8%)	0.66 (0.34, 1.26)				0.7 (0.36, 1.35)				0.7 (0.36, 1.36)			
Yes	Yes	70 (0.6%)	**0.49 (0.3, 0.82)**	0.9 (0.27, 4.16)	0 (-1.09, 1.1)	0.01 (-2.23, 2.24)	0.61 (0.36, 1.02)	0.97 (0.29, 4.52)	0.01 (-1.17, 1.19)	0.02 (-1.93, 1.97)	0.66 (0.37, 1.17)	0.98 (0.29, 4.57)	0 (-1.24, 1.24)	0 (-1.9, 1.9)
Calcium	Iron													
No	No	37 (1.1%)	1 (ref)				1 (ref)				1 (ref)			
No	Yes	0 (0%)	0.15 (0, 1.04)				0.17 (0, 1.22)				0.19 (0, 1.38)			
Yes	No	36 (0.7%)	0.68 (0.43, 1.07)				0.73 (0.46, 1.16)				0.82 (0.48, 1.41)			
Yes	Yes	37 (0.5%)	**0.5 (0.32, 0.79)**	5.02 (0.66, 643.79)	**0.68 (0.17, 1.18)**	1.35 (0.22, 2.48)	0.65 (0.42, 1.03)	5.22 (0.69, 670.88)	**0.75 (0.2, 1.31)**	1.15 (0.23, 2.07)	0.72 (0.42, 1.25)	4.66 (0.6, 600.11)	**0.72 (0.09, 1.34)**	0.99 (0.02, 1.96)
Calcium	Multivitamin													

Micronutrients			Crude model				Adjusted model [a]				Full-inclusion model [b]			
		N (%)	OR	IOR	RERI	AP	OR	IOR	RERI	AP	OR	IOR	RERI	AP
No	No	34 (1.1%)	1 (ref)				1 (ref)				1 (ref)			
No	Yes	37 (0.7%)	0.63 (0.39, 1.01)				0.68 (0.43, 1.1)				0.86 (0.49, 1.53)			
Yes	No	3 (0.5%)	0.47 (0.11, 1.31)				0.62 (0.15, 1.77)				0.71 (0.17, 2.09)			
Yes	Yes	36 (0.6%)	**0.53 (0.33, 0.85)**	1.79 (0.58, 7.93)	0.43 (-0.19, 1.06)	0.82 (-0.44, 2.07)	0.74 (0.46, 1.22)	1.75 (0.56, 7.75)	0.44 (-0.37, 1.24)	0.59 (-0.52, 1.7)	0.99 (0.53, 1.84)	1.62 (0.5, 7.29)	0.42 (-0.54, 1.37)	0.42 (-0.56, 1.41)
Folic acid	Iron													
No	No	22 (1.3%)	1 (ref)				1 (ref)				1 (ref)			
No	Yes	0 (0%)	0.24 (0, 1.77)				0.27 (0, 2.03)				0.28 (0, 2.15)			
Yes	No	51 (0.8%)	**0.57 (0.35, 0.96)**				0.62 (0.38, 1.04)				0.62 (0.34, 1.14)			
Yes	Yes	37 (0.5%)	**0.4 (0.24, 0.67)**	2.86 (0.37, 368.66)	0.58 (-0.14, 1.31)	1.47 (-0.57, 3.5)	**0.53 (0.31, 0.89)**	3.09 (0.4, 399.32)	0.63 (-0.15, 1.42)	1.2 (-0.43, 2.84)	0.52 (0.26, 1.04)	3.03 (0.38, 392.6)	0.62 (-0.17, 1.41)	1.19 (-0.58, 2.97)
Folic acid	Multivitamin													
No	No	21 (1.3%)	1 (ref)				1 (ref)				1 (ref)			
No	Yes	50 (0.7%)	**0.56 (0.34, 0.96)**				0.6 (0.36, 1.03)				0.66 (0.36, 1.22)			
Yes	No	1 (0.6%)	0.45 (0.03, 2.18)				0.55 (0.03, 2.71)				0.6 (0.03, 3.01)			
Yes	Yes	38 (0.6%)	**0.44 (0.26, 0.76)**	1.74 (0.34, 32.01)	0.43 (-0.52, 1.37)	0.97 (-1.29, 3.22)	0.62 (0.36, 1.09)	1.87 (0.36, 34.5)	0.47 (-0.68, 1.61)	0.75 (-1.16, 2.66)	0.73 (0.36, 1.46)	1.85 (0.35, 34.38)	0.47 (-0.77, 1.72)	0.65 (-1.14, 2.44)
Iron	Multivitamin													
No	No	60 (1%)	1 (ref)				1 (ref)				1 (ref)			
No	Yes	11 (0.4%)	**0.39 (0.2, 0.72)**				**0.47 (0.23, 0.87)**				**0.51 (0.25, 0.99)**			
Yes	No	13 (0.5%)	**0.51 (0.27, 0.9)**				0.67 (0.35, 1.2)				0.73 (0.37, 1.34)			
Yes	Yes	26 (0.6%)	**0.58 (0.36, 0.92)**	**2.9 (1.18, 7.63)**	**0.68 (0.24, 1.11)**	1.17 (0.4, 1.94)	0.83 (0.51, 1.34)	**2.6 (1.06, 6.88)**	**0.68 (0.11, 1.25)**	0.82 (0.17, 1.48)	0.91 (0.53, 1.56)	2.42 (0.97, 6.47)	**0.67 (0.05, 1.28)**	0.73 (0.07, 1.4)
							**Personal-social domain**							
Calcium	Folic acid													
No	No	50 (3.2%)	1 (ref)				1 (ref)				1 (ref)			
No	Yes	9 (3.1%)	0.95 (0.43, 1.86)				0.98 (0.44, 1.93)				1.01 (0.45, 2)			
Yes	No	58 (2.6%)	0.8 (0.55, 1.18)				0.79 (0.53, 1.16)				0.8 (0.54, 1.19)			
Yes	Yes	238 (2.1%)	**0.63 (0.46,**	0.83 (0.4, 1.91)	-0.12 (-0.86,	-0.19 (-1.34,	**0.69 (0.5, 0.95)**	0.89 (0.43, 2.07)	-0.08 (-0.84,	-0.11 (-1.2, 0.98)	0.72 (0.51,	0.89 (0.42, 2.06)	-0.09 (-0.87,	-0.13 (-1.2, 0.94)

Micronutrients		Crude model					Adjusted model [a]				Full-inclusion model [b]			
		N (%)	OR	IOR	RERI	AP	OR	IOR	RERI	AP	OR	IOR	RERI	AP
			**0.86)**		0.62)	0.96)			0.68)		1.02)		0.69)	
Calcium	Iron													
No	No	100 (2.9%)	1 (ref)				1 (ref)				1 (ref)			
No	Yes	8 (2.6%)	0.9 (0.4, 1.75)				0.91 (0.4, 1.79)				1.02 (0.45, 2.03)			
Yes	No	108 (2.2%)	**0.75 (0.57, 0.99)**				0.78 (0.59, 1.03)				0.9 (0.65, 1.24)			
Yes	Yes	139 (2%)	**0.7 (0.54, 0.9)**	1.04 (0.51, 2.42)	0.05 (-0.63, 0.73)	0.07 (-0.91, 1.06)	0.81 (0.62, 1.05)	1.14 (0.55, 2.67)	0.12 (-0.58, 0.82)	0.15 (-0.72, 1.02)	0.97 (0.7, 1.35)	1.06 (0.51, 2.51)	0.05 (-0.74, 0.85)	0.06 (-0.76, 0.87)
Calcium	Multivitamin													
No	No	90 (2.8%)	1 (ref)				1 (ref)				1 (ref)			
No	Yes	139 (2.5%)	0.89 (0.68, 1.17)				0.94 (0.71, 1.23)				1.04 (0.75, 1.45)			
Yes	No	18 (3%)	1.07 (0.62, 1.75)				1.22 (0.7, 2.01)				1.36 (0.77, 2.27)			
Yes	Yes	108 (1.7%)	**0.6 (0.45, 0.79)**	0.62 (0.36, 1.13)	-0.37 (-0.98, 0.24)	-0.62 (-1.62, 0.38)	**0.71 (0.53, 0.95)**	0.62 (0.35, 1.13)	-0.45 (-1.13, 0.24)	-0.63 (-1.58, 0.32)	0.78 (0.54, 1.12)	0.55 (0.31, 1.02)	-0.62 (-1.41, 0.18)	-0.8 (-1.77, 0.18)
Folic acid	Iron													
No	No	53 (3.1%)	1 (ref)				1 (ref)				1 (ref)			
No	Yes	6 (3.9%)	1.27 (0.48, 2.79)				1.37 (0.51, 3.03)				1.54 (0.57, 3.52)			
Yes	No	155 (2.3%)	0.73 (0.53, 1.01)				0.74 (0.54, 1.02)				0.82 (0.57, 1.19)			
Yes	Yes	141 (2%)	**0.63 (0.46, 0.87)**	0.68 (0.3, 1.85)	-0.37 (-1.48, 0.75)	-0.58 (-2.3, 1.15)	**0.72 (0.52, 1)**	0.72 (0.31, 1.95)	-0.38 (-1.58, 0.82)	-0.53 (-2.16, 1.11)	0.87 (0.57, 1.32)	0.69 (0.3, 1.88)	-0.5 (-1.87, 0.88)	-0.57 (-2.11, 0.97)
Folic acid	Multivitamin													
No	No	57 (3.4%)	1 (ref)				1 (ref)				1 (ref)			
No	Yes	172 (2.5%)	**0.71 (0.53, 0.97)**				**0.71 (0.53, 0.98)**				0.73 (0.51, 1.05)			
Yes	No	2 (1.1%)	0.33 (0.05, 1.06)				0.36 (0.06, 1.17)				0.37 (0.06, 1.22)			
Yes	Yes	124 (1.8%)	**0.53 (0.38, 0.73)**	2.27 (0.68, 14.08)	0.49 (-0.01, 0.99)	0.93 (-0.09, 1.94)	**0.61 (0.44, 0.84)**	2.37 (0.71, 14.77)	**0.54 (0, 1.08)**	0.88 (-0.07, 1.84)	**0.62 (0.42, 0.94)**	2.3 (0.68, 14.38)	0.52 (-0.04, 1.08)	0.84 (-0.15, 1.82)
Iron	Multivitamin													
No	No	160 (2.7%)	1 (ref)				1 (ref)				1 (ref)			
No	Yes	69 (2.5%)	0.93 (0.69, 1.23)				1.02 (0.76, 1.35)				1.13 (0.82, 1.55)			

Micronutrients		Crude model					Adjusted model [a]				Full-inclusion model [b]			
		N (%)	OR	IOR	RERI	AP	OR	IOR	RERI	AP	OR	IOR	RERI	AP
Yes	No	48 (1.9%)	**0.7 (0.5, 0.96)**				0.8 (0.57, 1.11)				0.88 (0.61, 1.23)			
Yes	Yes	78 (1.8%)	**0.65 (0.49, 0.85)**	0.99 (0.63, 1.59)	0.02 (-0.35, 0.38)	0.03 (-0.54, 0.59)	0.78 (0.59, 1.04)	0.96 (0.61, 1.54)	-0.03 (-0.45, 0.38)	-0.04 (-0.57, 0.49)	0.88 (0.64, 1.21)	0.89 (0.56, 1.44)	-0.13 (-0.59, 0.34)	-0.14 (-0.68, 0.39)

[a] Adjusted Model: Adjusted for child's basic characteristics, maternal demographic characteristics, pregnancy and perinatal characteristics and childhood family environment.

[b] Full-inclusion Model: Included all micronutrients in the model and adjusted for child's basic characteristics, maternal demographic characteristics, pregnancy and perinatal characteristics and childhood family environment.
