# Supplementary material for: The Individual and Combined Effects of Prenatal Micronutrient Supplementations on Neurobehavioral Developmental Disorders in Preschool Children
Source: Children (Basel). 2025 May 5;12(5):602. doi: 10.3390/children12050602 (PMC12110273; doi:10.3390/children12050602)
Supplement: Supplementary file 1 [file children-12-00602-s001.zip › Text S1 Sample Size Calculation.pdf]

Supplementary Text 1 Sample Size Calculation

This study is based on a large-scale cross-sectional child health survey. To ensure the adequacy of the sample for statistical analyses, we performed sample size estimations using two approaches:

(1) Sample Size Estimation Based on a Cross-Sectional Survey Design

The minimum required sample size for estimating the prevalence of neurobehavioral developmental problems was calculated using the following formula:

$$\mathrm{N} = \frac{Z_{1-\alpha/2}^{2} \times pq}{d^2}$$

Where:

- p represents the expected prevalence (15.6%, based on Shenzhen child data from 2015 [42])
- q=1−p
- d is the allowable error (set at 15% of p)
- α=0.05(two-sided test)
- $Z_{1-\alpha/2}$=1.96

This calculation yielded a minimum sample size of 924 participants for estimating the prevalence of neurobehavioral developmental problems.

(2) Sample Size Estimation Based on Logistic Regression Analysis of Maternal Micronutrient Supplementation and Child Neurobehavioral Development

To estimate the sample size required for logistic regression analysis, we used the following formula:

$$\mathrm{N} = \frac{\{Z_{1-\alpha/2}[p(1-p)/B]^{1/2} + Z_{1-\beta}[p_0(1-p_0) + p_1(1-p_1)(1-B)/B]^{1/2}\}^2}{(p_0-p_1)^2(1-B)}$$

where:

- α=0.10 is the significance level (two-sided), and $Z_{1-\alpha/2}$ is the corresponding

standard normal value

- β=0.20 corresponds to a power of 80%, and $Z_{1-\beta}$ is the standard normal value at this power level
- p=0.156 represents the overall prevalence of neurobehavioral developmental problems among children, based on 2015 Shenzhen data [42];
- B is the proportion of pregnant women who received supplementation with a given micronutrient;
- p_0 is the rate of neurobehavioral problems in children whose mothers did not receive the supplement;
- p_1 is the rate in those whose mothers did receive the supplement.

Based on preliminary analysis of 1,000 participants, the estimated values were as follows:

- For calcium: B=75.8%, p_0 =11.7%, p_1=9.9%
- For folic acid: B=88.4%, p_0 0=11.5%, p_1=9.3%
- For iron: B=45.6%, p_0 =11.8%, p_1=10.1%
- For multivitamins: B=45.0%, p_0 =12.5%, p_1=10.0%

Based on these parameters, the estimated minimum sample sizes required for detecting the respective associations were: 13,457 for calcium, 15,531 for folic acid, 11,976 for iron, and 5,620 for multivitamins.

To meet the most stringent requirement, a minimum of 15,531 participants was needed. Ultimately, 15,636 children were included in this study, confirming that the final sample was sufficient to support the planned analyses.

Reference

42. W., D.; H., W.; J., C. Analysis of developmental screening results using ASQ-3 among 2,246 infants aged 3 to 4 months in Shenzhen (in Chinese). *J Huazhong Univ Sci Technol Med Sci* **2016**, *045*, 454-457.
